# Non-Toxic Metabolic Management of Metastatic Cancer in VM Mice: Novel Combination of Ketogenic Diet, Ketone Supplementation, and Hyperbaric Oxygen Therapy

**DOI:** 10.1371/journal.pone.0127407

**Published:** 2015-06-10

**Authors:** A. M. Poff, N. Ward, T. N. Seyfried, P. Arnold, D. P. D’Agostino

**Affiliations:** 1 Department of Molecular Pharmacology and Physiology, Morsani College of Medicine, Hyperbaric Biomedical Research Laboratory, University of South Florida, Tampa, Florida, United States of America; 2 Department of Biology, Boston College, Chestnut Hill, Massachusetts, United States of America; 3 Savind, Inc. Seymour, Illinois, United States of America; Roswell Park Cancer Institute, UNITED STATES

## Abstract

The Warburg effect and tumor hypoxia underlie a unique cancer metabolic phenotype characterized by glucose dependency and aerobic fermentation. We previously showed that two non-toxic metabolic therapies – the ketogenic diet with concurrent hyperbaric oxygen (KD+HBOT) and dietary ketone supplementation – could increase survival time in the VM-M3 mouse model of metastatic cancer. We hypothesized that combining these therapies could provide an even greater therapeutic benefit in this model. Mice receiving the combination therapy demonstrated a marked reduction in tumor growth rate and metastatic spread, and lived twice as long as control animals. To further understand the effects of these metabolic therapies, we characterized the effects of high glucose (control), low glucose (LG), ketone supplementation (βHB), hyperbaric oxygen (HBOT), or combination therapy (LG+βHB+HBOT) on VM-M3 cells. Individually and combined, these metabolic therapies significantly decreased VM-M3 cell proliferation and viability. HBOT, alone or in combination with LG and βHB, increased ROS production in VM-M3 cells. This study strongly supports further investigation into this metabolic therapy as a potential non-toxic treatment for late-stage metastatic cancers.

## Introduction

Cancers exhibit a dysregulated metabolic phenotype characterized by lactate fermentation in the presence of oxygen, a phenomenon known as the Warburg effect [1]. Interest in cancer metabolism has increased over the past decade, with researchers suggesting numerous causes and consequences of the Warburg effect, and investigating novel therapeutic strategies to exploit it [1–5]. Glucose dependency and lactate production, two key features of the Warburg effect, correlate strongly with aggressive capacity and invasive potential [3, 4, 6, 7]. Metastasis, or the spread of tumor cells from a primary site to distal tissues, is responsible for over 90 percent of cancer-related deaths. There are no cancer therapies currently available that can effectively manage systemic metastasis. Since the Warburg effect is a prominent phenotype in metastatic cells, metabolic therapies which exploit this phenomenon may offer new therapeutic options for patients with aggressive or late-stage cancers.

The glycolytic-dependency associated with the Warburg effect has led researchers to investigate dietary therapies which decrease glucose availability to the tumor. The ketogenic diet (KD) is a high fat, adequate protein, very low carbohydrate diet which has been used in preclinical and clinical studies to slow cancer progression [8–21]. The KD forces a physiological shift to fat metabolism, lowing blood glucose, suppressing insulin, and elevating blood ketone levels by stimulating ketogenesis from dietary and stored fats. Although the anti-cancer effects of the KD are largely attributed to a reduction in the glycolytic substrates and insulin signaling which fuel cancer metabolism, emerging evidence suggests that ketones have a therapeutic potential of their own [17, 22, 23]. While healthy cells readily adapt to ketones as an efficient energy substrate, many cancers are not able to make this adaptation [24, 25].

The expression of ketone utilization enzymes is often reduced in malignant cancers compared to their normal tissue counterparts [26, 27]. Indeed, unlike in healthy neurons, ketones fail to rescue glioma cells from glucose deprivation-induced death [28]. We recently described this phenomenon and investigated the potential use of dietary ketone supplementation in a mouse model of metastatic cancer [22]. Our study showed that exogenous ketone supplementation exerts potent anti-cancer effects *in vivo*, even when administered with a high carbohydrate diet, with similar effects on cancer cells in the presence of high glucose media *in vitro* [22].

Cellular energy metabolism is intricately linked to the oxygenation status of the tissue [29, 30]. When oxygen is readily available, normal cells produce up to 90 percent of their ATP by mitochondrial respiration. When oxygen availability becomes limited, such as in the exercising muscle, cells convert to using anaerobic fermentation to preserve ATP production. This metabolic switch sustains cellular function and promotes survival in the face of transient hypoxia, and its mechanism is largely driven by the HIF-1 transcription factor [30, 31]. HIF-1 enhances the expression of over 60 genes, many involved in glycolysis and fermentation, angiogenesis, growth, and survival [30, 32]. The aberrant signaling that drives tumor angiogenesis creates immature and leaky blood vessels which are unable to adequately perfuse the entire tumor [33]. This leads to the formation of hypoxic regions inside the tumor which enhance the Warburg effect and promote cancer progression, invasion, and metastasis [33–35]. Tumor hypoxia and HIF-1 signaling are both strongly correlate with aggressive capacity and poor prognosis [36]. Tumor hypoxia is also known to mediate some chemo- and radio-resistance [37–40]. Because these therapies work in large part by stimulating the overproduction of reactive oxygen species (ROS) within the tumor, limited oxygen availability lessens their efficacy [41, 42].

Hyperbaric oxygen therapy (HBOT) is the administration of 100% oxygen at elevated pressure. *In vivo*, HBOT saturates blood plasma with oxygen, allowing it to diffuse further into the tissues and oxygenate hypoxic tumor regions [43–45]. Similarly, HBOT increases oxygen diffusion into cells in culture and thus its effects can be readily evaluated in the *in vitro* environment. HBOT has been shown to inhibit angiogenesis and tumor growth and increase survival time as a stand-alone or adjuvant therapy to standard care in a variety of cell, animal, and human studies [46–52]. We previously showed that the ketogenic diet with concurrent hyperbaric oxygen therapy was an effective combination therapy against metastatic cancer [9].

The ketogenic diet, ketone supplementation, and HBOT target overlapping metabolic pathways which are especially prominent in metastatic cells. We hypothesized that combining these three metabolic therapies could provide a safe, cost-effective adjuvant treatment for metastatic cancer. We tested this hypothesis *in vivo* and *in vitro* using the VM-M3 mouse model of metastatic cancer [9, 22, 53].

## Materials and Methods

### Cell Culture

VM-M3/Fluc cells (VM-M3) were acquired as a gift from T. Seyfried of Boston College where they were obtained from a spontaneous brain tumor in a VM/Dk inbred mouse, adapted to cell culture, and transduced with a lentivirus vector containing firefly luciferase under control of the cytomegalovirus promoter as previously described [53]. VM-M3 cells were cultured in Dulbecco’s Modified Eagle Medium (Gibco, Life Technologies) with 5mM L-glutamine (ATCC), 10% fetal bovine serum (Invitrogen), 1% penicillin-streptomycin (Invitrogen), 10mM HEPES buffer (Gibco, Life Technologies), and high glucose (25mM D-glucose; Fisher Scientific). Cells were maintained in a humidified incubator at 37°C in 95% air and 5% CO_2_.

### Mice

Breeding pairs of the VM/Dk inbred strain of mice were received as a gift from T. Seyfried of Boston College, and were used to establish and propagate a mouse colony in the University of South Florida Morsani College of Medicine Vivarium according to standard husbandry protocol. Male mice (8–20 weeks of age) were used in the described study. All procedures were approved by the University of South Florida Institutional Animal Care and Use Committee (USF IACUC; Protocol Number R4137) and performed under strict adherence to the NIH Guide for the Care and Use of Laboratory Animals.

### VM-M3 Cell Implantation

Approximately 1 million VM-M3 cells in 300 μL sterile PBS were implanted subcutaneously into the lateral abdominal flank of VM/Dk mice using a 27g needle as previously described [9]. VM-M3 cell inoculation from this site results in rapid and systemic metastasis [9].

### Dietary Administration

Animals were randomly assigned to a study group and began receiving their respective diets on the day of tumor inoculation in order to maximize the therapeutic window in the highly aggressive VM-M3 model. Prior to the first day of the study, all mice were fasted overnight to encourage rapid compliance with the dietary protocol. Control animals received standard rodent chow (2018 Teklad Global 18% Protein Rodent Diet, Harlan) *ad libitum* for the duration of the study. KD+HBOT mice received KD-USF, a custom ketogenic diet designed by the authors and produced by Harlan Laboratories, fed *ad libitum*. KD+KE and KD+KE+HBOT mice received KD-USF mixed in with KE at 10% by volume with 1% saccharin for palatability. KE was synthesized in collaboration with Patrick Arnold (Savind Inc., Seymour, IL) as previously described [54]. The macronutrient profile and caloric density of SD, KD-USF, and the KE are shown in Table 1. All diets were monitored continuously and replaced twice weekly or as needed to maintain freshness for the duration of the study.

**Table 1:** Macronutrient information of diets and ketone ester.

Macronutrient Information	Standard Diet (SD)	Ketogenic Diet (KD)	Ketone Ester (KE)
% kcal from Fat	18.0	77.1	NA
% kcal from Protein	24.0	22.4	NA
% kcal from Carbohydrate	58.0	0.5	NA
Caloric Density	3.1 Kcal/g	4.7 Kcal/g	5.58 Kcal/g

### Hyperbaric Oxygen Therapy

KD+KE+HBOT mice received 90 minute hyperbaric oxygen sessions of 100% O_2_ at 2.5 ATA (1.5 ATM gauge) three times weekly (M,W,F) in a standard hyperbaric chamber (model 1300B, Sechrist Industries) for the duration of the study. KD+KE+HBOT received their first HBOT session 24 hours after tumor cell inoculation.

### Blood and Weight Measurements

Blood (approximately 10 μl) was collected once weekly from the tail using IACUC-approved methods. Blood glucose and β-hydroxybutyrate (βHB) was measured using the Precision Xtra Blood Glucose & Ketone Monitoring System (Abbott Laboratories). Mice were weighed in the mid-afternoon, twice weekly for the duration of the study using the AWS-1KG Portable Digital Scale (AWS).

### Bioluminescent Imaging and Tumor Growth Analysis

Bioluminescence of the luciferase-tagged VM-M3 cells *in vivo* was acquired using the IVIS Lumina cooled CCD camera system and Living Image software (Caliper LS). Fifteen minutes prior to imaging, mice received an i.p. injection of 50 mg/kg D-Luciferin (Caliper LS). Whole animal bioluminescent signal (photons/sec) was measured once weekly as an indicator of metastatic tumor size and spread. Tumor take and metastatic spread was confirmed in all animals within 14 days following VM-M3 cell inoculation by bioluminescent imaging. *In vivo* bioluminescent signal (photons/sec) of key metastatic regions (brain, lungs, and liver) was measured as an indicator of metastatic tumor burden and spread at 21 days post inoculation in the survival study animals [43, 55]. Extent of metastatic spread was further analyzed in a separate group of control and combination therapy treated animals with *ex vivo* organ bioluminescent imaging. Animals were inoculated with VM-M3 cells, treated for 21 days, and humanely euthanized. Tissues were harvested and placed in a petri dish containing 300 ug/mL luciferin in sterile PBS for 5 minutes prior to acquisition of bioluminescent signal.

### Survival Analysis

Physical and behavioral health of the mice were assessed daily throughout the study. Mice were humanely euthanized by CO_2_ asphyxiation according to approved IACUC guidelines upon presentation of defined criteria associated with disease progression (abnormal feeding behavior, tumor-associated ascites, decreased response to stimuli, failure to thrive).

### Immunohistochemical Analysis of Tumor Vascularization

After 21 days of treatment, animals in the aforementioned 3-week treatment study were humanely euthanized and livers were harvested and preserved in 10% neutral, phosphate buffered formalin. The liver tissue was paraffin-embedded, cut into 5 micron sections, and mounted onto microscope slides at the USF Morsani College of Medicine Histology Core. One set of slides was stained with hematoxylin and eosin and mounted with cover slips for morphological analysis. Another set of slides was probed for the endothelial cell marker CD31 to visualize blood vessels. Slides were rehydrated through a series of xylene and ethanol washes, and antigen was retrieved by a standard heat-mediated citrate buffer method. Slides were incubated in 0.3% H_2_O_2_ in dH_2_O to inhibit endogenous peroxide activity. Immunohistochemical analysis of the slides was performed using the VECTASTAIN Elite ABC kit. Slides were blocked for 30 minutes in normal blocking serum, and then incubated in anti-CD31 antibody (1:25; ab28364, Abcam) for one hour at room temperature. Slides were then incubated in biotinylated anti-rabbit secondary antibody in normal blocking serum for 30 minutes at room temperature, followed by VECTASTAIN ABC reagent containing avidin and biotinylated horseradish peroxidase solution for 30 minutes at room temperature. Slides were developed using the VECTOR NovaRED Peroxidase (HRP) Substrate Kit. Slides were counterstained with hematoxylin, washed, mounted with coverslips, and visualized with light microscopy. At least 3 frames containing tumor tissue per sample were analyzed. The number of CD31^+^ blood vessels associated with tumors in each frame was counted and compared between control and treated groups as a measure of tumor vascularization.

### VM-M3 Cell Proliferation

50,000 VM-M3 cells were plated in 35 mm 6 well plates in 1 mL of high glucose media (control; 25mM) or low glucose media (LG; 3 mM) with or without daily HBO treatments (HBOT; 100% O_2_, 90 min, 2.5 ATA). A combination treatment group received low glucose, ketone supplementation (5mM βHB), and daily HBO treatments (LG+βHB+HBOT). These studies were performed in tandem with work investigating the effect of ketone supplementation on VM-M3 cell proliferation; therefore, the 25mM and 3mM glucose proliferation curves presented are the same as previously reported [22]. Cells were grown for 24, 48, 72, and 96 hours to determine a growth curve. Media was replaced in all treatments at 48 hours, and each time point and treatment group was run in triplicate. Cells were scraped and counted using standard trypan blue hemocytometry. Plates were visually inspected to ensure all cells were harvested prior to counting.

### VM-M3 Cell Viability

Viability was assessed with the LIVE/DEAD Viability/Cytotoxicity Kit (Invitrogen). 50,000 VM-M3 cells were plated into 22 mm 12 well plates containing Poly-D-Lysine coated glass coverslips (BrainBits) in 1mL control media and allowed to grow for 24 hrs. At 24 hrs, cells were administered treatment: high glucose media (control; 25mM), low glucose media (LG; 3mM), control media (25mM glucose) with a single session of hyperbaric oxygen (HBOT; 100% O_2_, 90 min, 2.5 ATA), or the combination therapy of low glucose, ketone supplementation (5mM βHB), and a single session of hyperbaric oxygen (LG+βHB+HBOT). These studies were performed in tandem with work investigating the effect of ketone supplementation on VM-M3 cell viability; therefore, the 25mM and βHB viability data are the same as previously reported and are shown here for reference [22]. At 48 hours, cells were rinsed with D-PBS and incubated in 2μM calcein AM (Ex/Em: 495/515) and 4μM EthD-1 (Ex/Em: 525/590) in D-PBS for 30 min at 37°C. Coverslips were inverted, mounted, and sealed on a microscope slide, and cells were visualized with a Nikon TE200E using fluorescence confocal microscopy with FIT-C and TRIT-C filters to identify live and dead cells, respectively. Treatments were run in triplicate. The numbers of live and dead cells were counted in 10 frames per coverslip using a 10x objective to determine percent viability.

### VM-M3 Cell ROS Detection

50,000 VM-M3 cells were plated in 35 mm Poly-D-Lysine coated glass FluoroDish tissue culture dishes (World Precision Instruments) in 1 mL control media and allowed to grow for 24 hours. At 24 hrs, cell were administered treatment: media was replaced with high glucose media (control; 25mM), low glucose media (LG; 3mM), control media with ketone supplementation (βHB; 5mM βHB), control media with a single session of hyperbaric oxygen (HBOT; 100% O2, 90 min, 2.5 ATA), or combination therapy (LG+βHB+HBOT). At 48 hours, cells were incubated in 10μM DHE (Ex/Em: in CSF for 30 min at 37°C, and superoxide production was measured using fluorescence confocal microscopy with a TRIT-C filter as previously described [56]. Treatments were run in replicate. Superoxide production was measured by calculating the average of the fluorescence intensity units (FIU) per cell in 10 frames per sample using a 10x objective.

### Statistics

Survival was analyzed with the Kaplan-Meier and log-rank Mantel-Cox tests for survival distribution. Cell viability and tumor vascularization were analyzed by unpaired t test. Bioluminescent signal was analyzed by Mann-Whitney U test. Cell viability, cell proliferation, mean survival, blood glucose and βHB, and percent body weight change were analyzed by one-way ANOVA with Tukey’s multiple comparison test post hoc. All statistical analysis was performed using GraphPad Prism 6 software. Results were considered significant when p<.05. The data for this study is available as supplemental information (S3 File).

## Results

### Metabolic therapy slows tumor growth, inhibits metastatic spread and tumor vascularization, and extends survival time in mice with metastatic cancer

**Fig 1:**
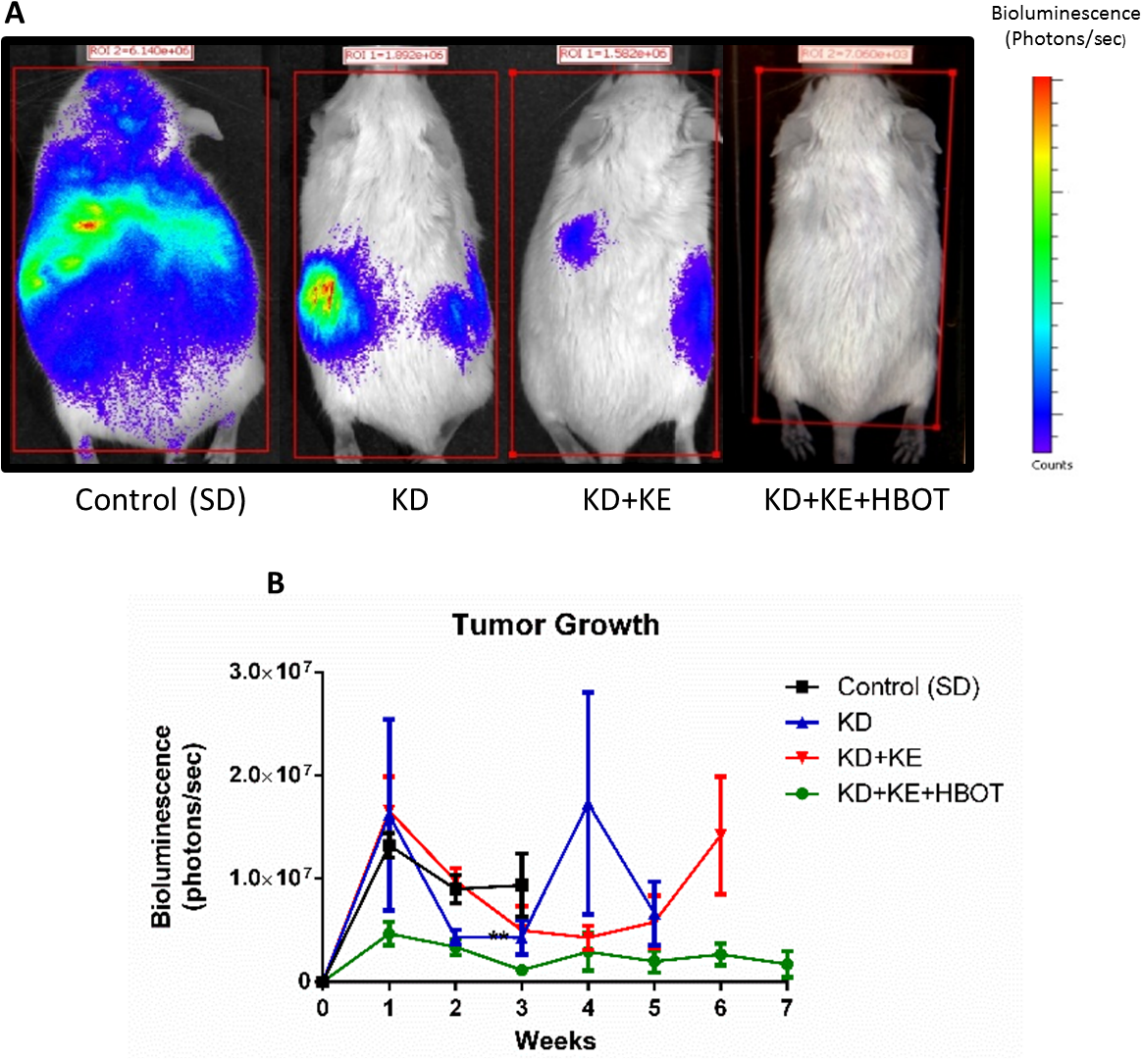
Combination metabolic therapy slows tumor growth. **(A)** *In vivo* bioluminescence imaging of representative animals from each treatment group 3 weeks post tumor cell inoculation. Treated mice exhibited reduced tumor burden and metastatic spread. **(B)** Whole animal bioluminescence (photons/sec) was tracked over time as a measure of tumor growth rate. In order to avoid misleading representation of tumor burden, data for treatment groups where ≥50% of animals had succumbed to disease progression are no longer shown. Tumor bioluminescence was highly variable, but treated animals demonstrated a notable trend of slower tumor growth compared to controls throughout the study. KD treated mice had less bioluminescence than controls at week 2, while KD+KE+HBOT mice had less bioluminescence than control animals at weeks 1, 2, and 3. KD+KE+HBOT mice also had less bioluminescence than KD+KE mice at weeks 1, 2, 3, and 5, and had less tumor bioluminescence than KD mice at weeks 3 and 5. Error bars represent **±**SEM. Results were considered significant when p<0.05.

**Fig 2:**
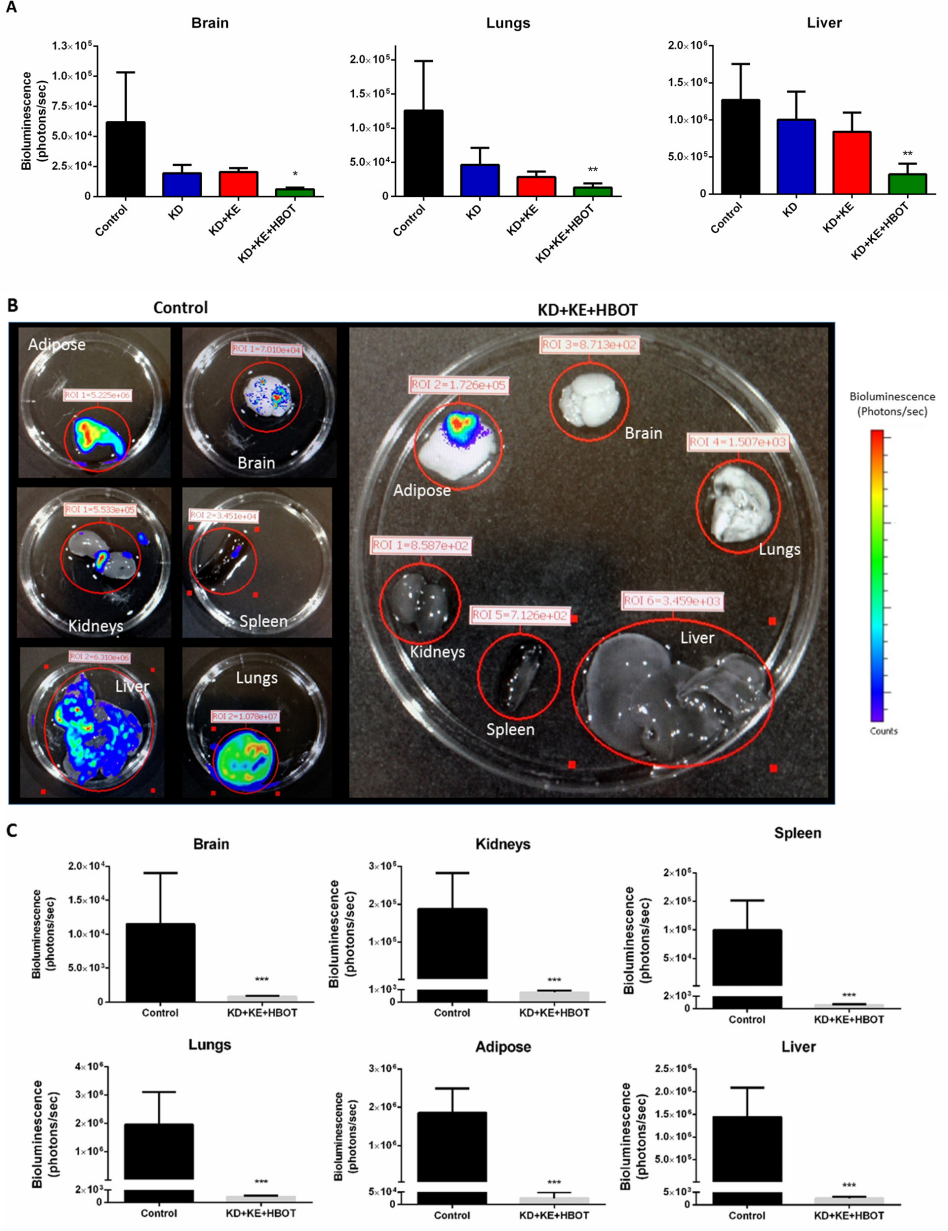
Combination metabolic therapy slows tumor growth and inhibits metastatic spread. **(A)** *In vivo* bioluminescence of key metastatic regions 21 days after tumor cell inoculation demonstrated a marked reduction in metastatic tumor burden and spread for treated animals. KD+KE+HBOT mice had significantly less metastatic spread to the brain, lung, and liver compared to controls at 3 weeks. There were no significant differences in metastatic spread between the three treatment groups. **(B-C)** To provide a more sensitive measure of metastatic spread in our multi-combination therapy, a separate group of control and KD+KE+HBOT treated animals were treated for 21 days following tumor cell inoculation and euthanized. *Ex vivo* bioluminescent imaging of harvested organs confirmed a significant decrease in metastatic tumor burden and spread in combination treated animals compared to controls. All organs tested—brain, kidneys, spleen, lungs, adipose, and liver—exhibited significantly reduced bioluminescent signal compared to controls. Error bars represent **±**SEM. Results were considered significant when p<0.05.

**Fig 3:**
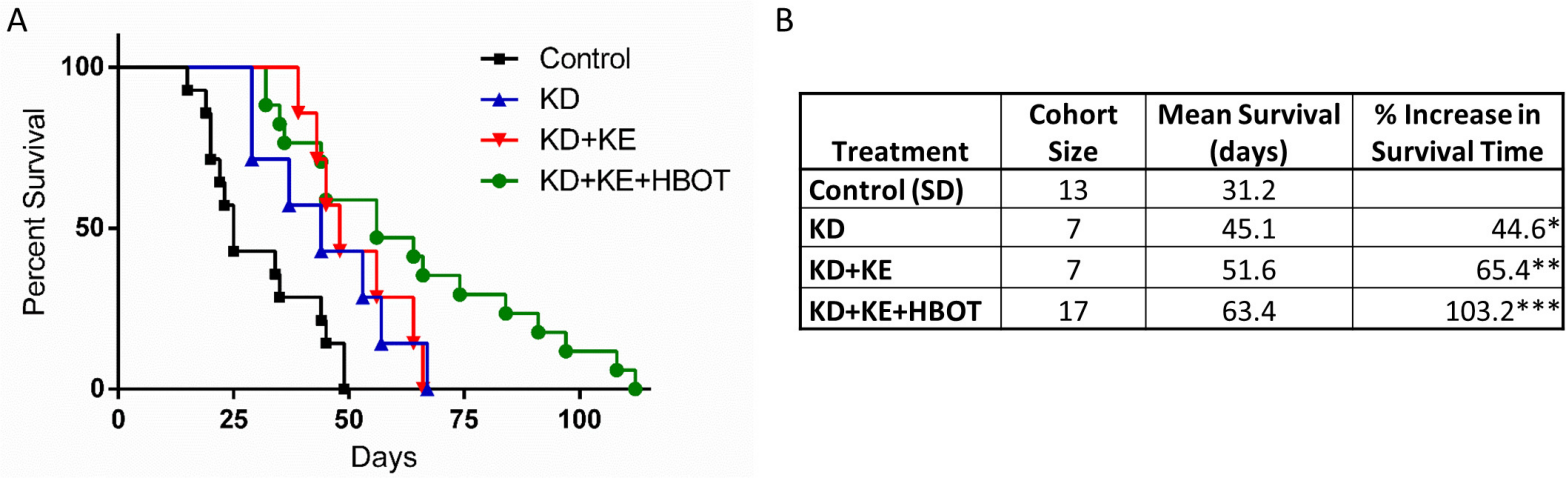
Combination metabolic therapy extends survival time in mice with metastatic cancer. **(A)** Kaplan-Meier survival curve of study groups. KD, KD+KE, and KD+KE+HBOT treated mice demonstrated prolonged survival compared to controls (p = 0.03, p = 0.009, and p<0.0001, respectively; log-rank Mantel-Cox test for survival distribution). There was a strong trend of more prolonged survival in the combination treatment mice compared to KD or KD+KE, but these curves were not significantly different from each other (KD vs. KD+KE+HBOT p = 0.0622; KD+KE vs. KD+KE+HBOT p = 0.1924; log-rank Mantel-Cox test). **(B)** Cohort size (N), mean survival (days), and percent increase in mean survival time from control. KD, KD+KE, and KD+KE+HBOT treated mice exhibited a 44.6%, 65.4%, and 103.2% increase in mean survival time compared to controls, respectively (*p<0.05, **p<0.01, ***p<0.001; One-Way ANOVA). Similar to the survival curve analysis, there was a trend of increased mean survival time in combination treated mice compared to KD and KD+KE, but these differences were not significant (KD vs. KD+KE+HBOT p = 0.1062; KD+KE vs. KD+KE+HBOT p = 0.2763). Results were considered significant when p<0.05.

**Fig 4:**
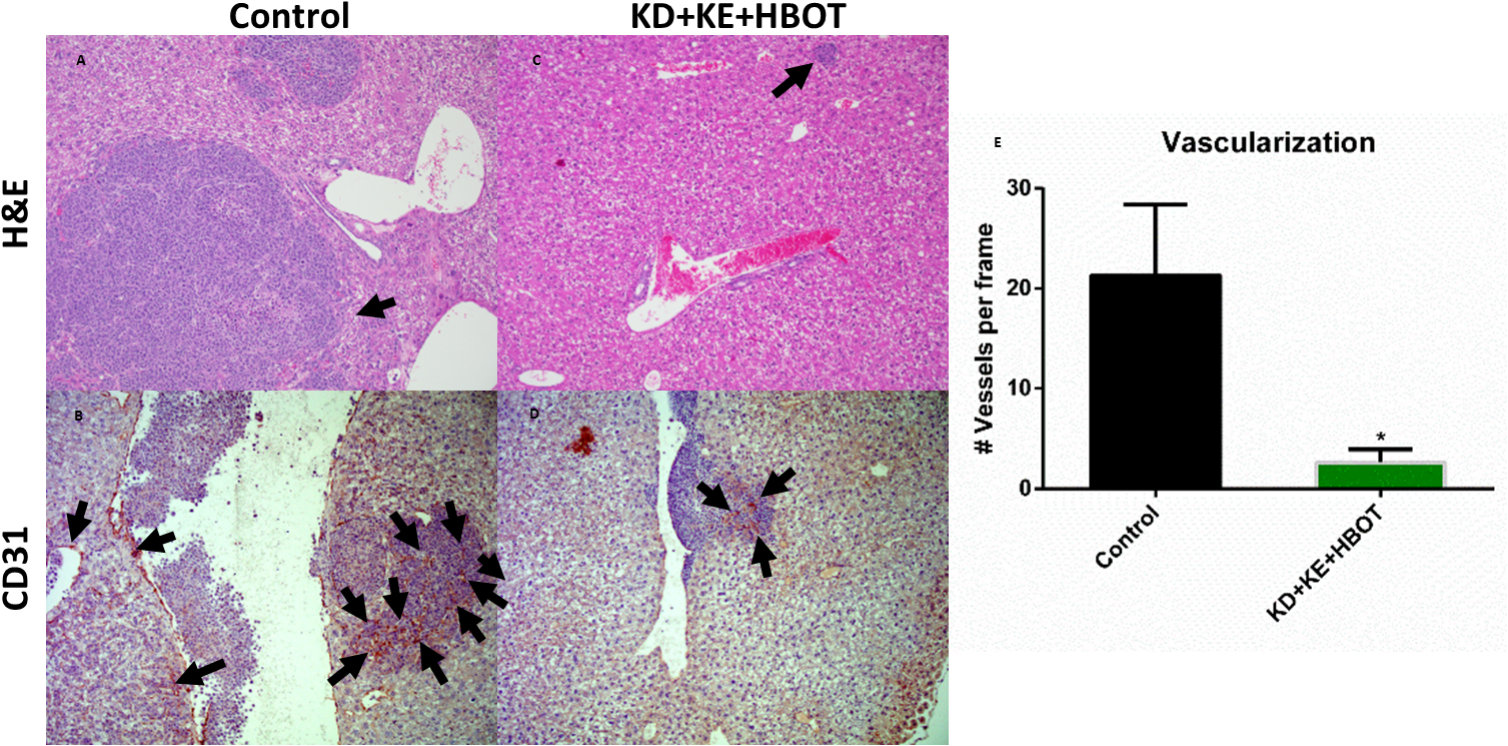
Combination metabolic therapy decreases metastatic liver tumor size and vascularization. Morphology and vascularization of KD+KE+HBOT-treated mouse liver tumors were analyzed by H&E staining and CD31 immunohistochemistry. Tumors from KD+KE+HBOT-treated mice were smaller with more well-defined borders (A, C) and exhibited a significant reduction in vascularization (B, D, E) from controls (*p<0.05; unpaired t-test). Error bars represent ±SEM.

**Fig 5:**
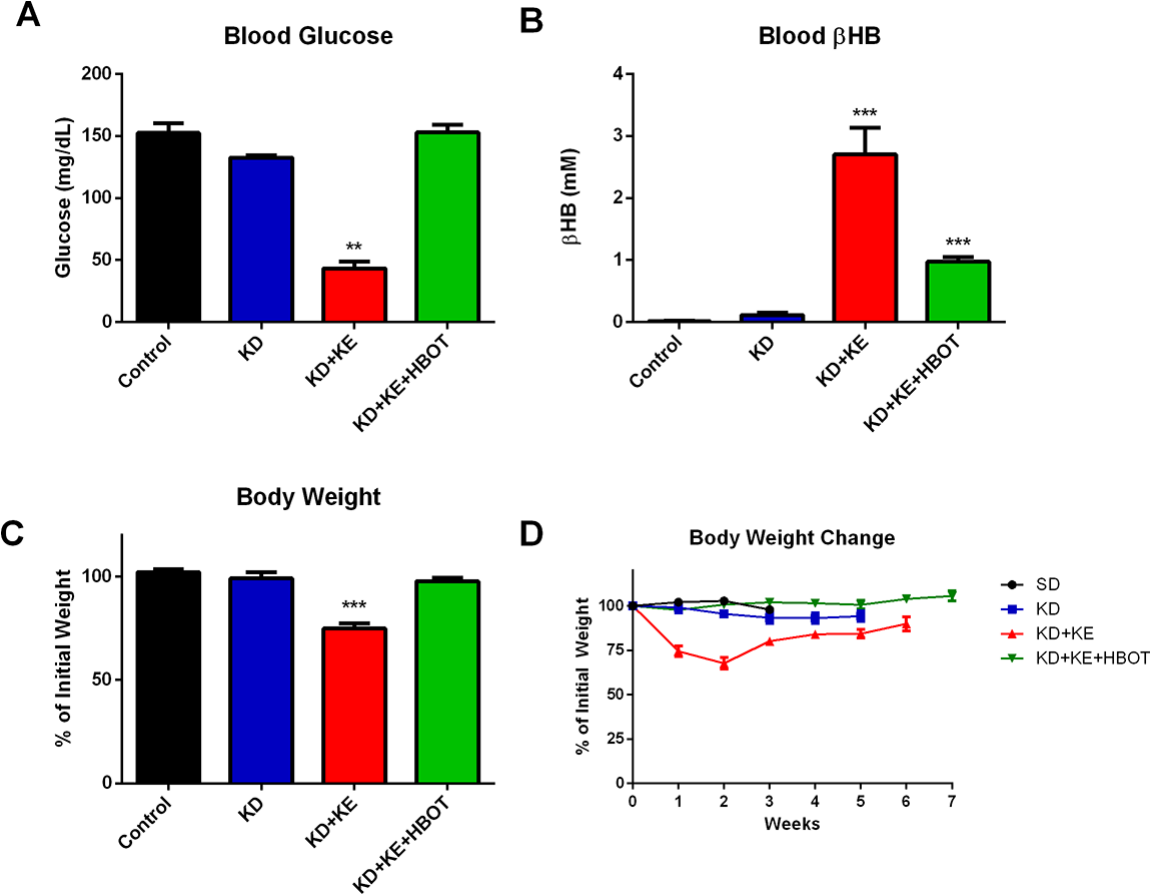
Blood glucose, βHB, and body weight of VM-M3 survival study mice prior to significant disease onset (day 7). **(A-C)** KD+KE treated mice had significantly decreased blood glucose and body weight and increased blood βHB compared to control mice by day 7. Blood glucose was significantly reduced only in KD+KE treated mice by day 7. Both KD+KE and KD+KE+HBOT treated mice exhibited increased blood βHB compared to control and KD treated mice by day 7. Only KD+KE mice had a significant reduction in body weight by day 7. **(D)** Only KD+KE mice demonstrated a significant and consistent decrease in body weight over the course of the study. Results were considered significant when p<0.05. Error bars represent **±**SEM.

Bioluminescent imaging showed a trend of reduced tumor growth rate and metastatic spread in treated animals compared to controls over the course of the study (Figs 1 and 2). Tumor bioluminescence was below detectable threshold in several KD+KE+HBOT mice at 3 weeks (Fig 1). Tumor take and spread from the site of inoculation was observed with bioluminescent imaging in all mice by day 14, indicating that these metabolic therapies did not prolong survival simply by preventing tumor formation. However, tumor bioluminescence was decreased in the triple-treatment mice compared to control at weeks 1 and 2, suggesting that the physiological environment induced by our metabolic therapy was less conducive to tumor growth and may have slowed growth of the primary tumor as well as metastatic spread. KD+KE+HBOT treated mice exhibited less overall tumor burden as measured with total bioluminescent signal than controls at all applicable time points (weeks 1, 2, and 3; Fig 1). KD+KE+HBOT treated mice also exhibited less overall tumor burden than KD mice on weeks 3 and 5 and KD+KE mice on weeks 1, 2, 3, and 5 (Fig 1). At 3 weeks post VM-M3 cell inoculation, KD+KE+HBOT treated mice had a significantly smaller overall tumor burden and less metastatic spread to the brain, lungs, liver, kidneys, spleen, and adipose tissue compared to control mice (***p<0.001, one-way ANOVA; Fig 1). KD, KD+KE, and KD+KE+HBOT mice demonstrated significantly prolonged survival curves compared to control animals (p = 0.03, p = 0.009, and p<0.0001, log-rank Mantel-Cox test for survival distribution; Fig 3A). KD treatment increased mean survival time by approximately 14 days (44.6%), KD+KE treatment by approximately 20 days (65.4%), and KD+KE+HBOT treatment by approximately 32 days (103.2%) compared to controls (*p<0.05; one-way ANOVA; Fig 3B). There were no significant differences in survival between the three treatment groups. Morphological and immunohistochemical analysis of livers from KD+KE+HBOT treated mice revealed only micrometastatic lesions with little notable vascularization compared to controls (Fig 4). Blood glucose and body weight decreased and blood βHB increased in mice receiving KD+KE therapy by day 7, prior to the onset of significant disease progression (Fig 5).

### Metabolic therapy decreases proliferation and viability and increase ROS production in VM-M3 cells

**Fig 6:**
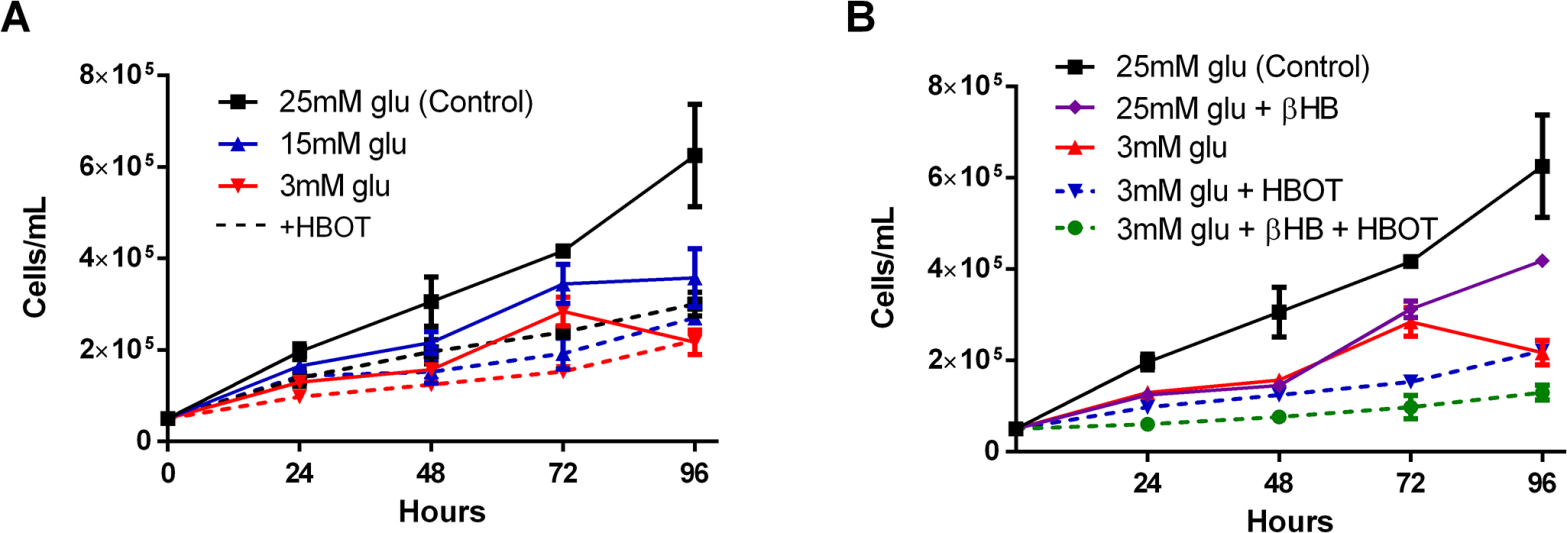
Combination metabolic therapy decreases VM-M3 cell proliferation *in vitro*. VM-M3 cells were grown in conditions mimicking metabolic therapy, including combinations of varied glucose concentration (25mM, 15mM, or 3mM) media, ketone supplementation (5mM βHB), and daily HBOT sessions (100% O_2_, 2.5 ATA, 90 min). Cell density was measured with trypan blue hemocytometry to produce a growth curve over 96 hours. **(A)** Proliferation of VM-M3 cells was inhibited with decreasing glucose concentration and the addition of HBOT. At 24 hours, cell density of 3mM glu and 3mM glu + HBOT cells was significantly less than control (25mM) glucose treated cells. At 48, 72, and 96 hours, the cell density of all treatment groups was significantly reduced compared to control (*p<0.05; Two-way ANOVA). **(B)** Combining low glucose, ketone supplementation, and HBOT resulted in a marked decrease in cell proliferation which was significant from control at all time points tested. (p<0.05; one-way ANOVA). Cells receiving combination therapy had a significant decrease in proliferation compared to cells treated with LG only at 24, 48, and 72 hours, and compared to βHB only at 48, 72, and 96 hours, and exhibited a non-significant trend of decreased proliferation compared to LG + HBOT treated cells for the duration of the growth curve. Results were considered significant when p<0.05. Error bars represent **±**SEM.

**Fig 7:**
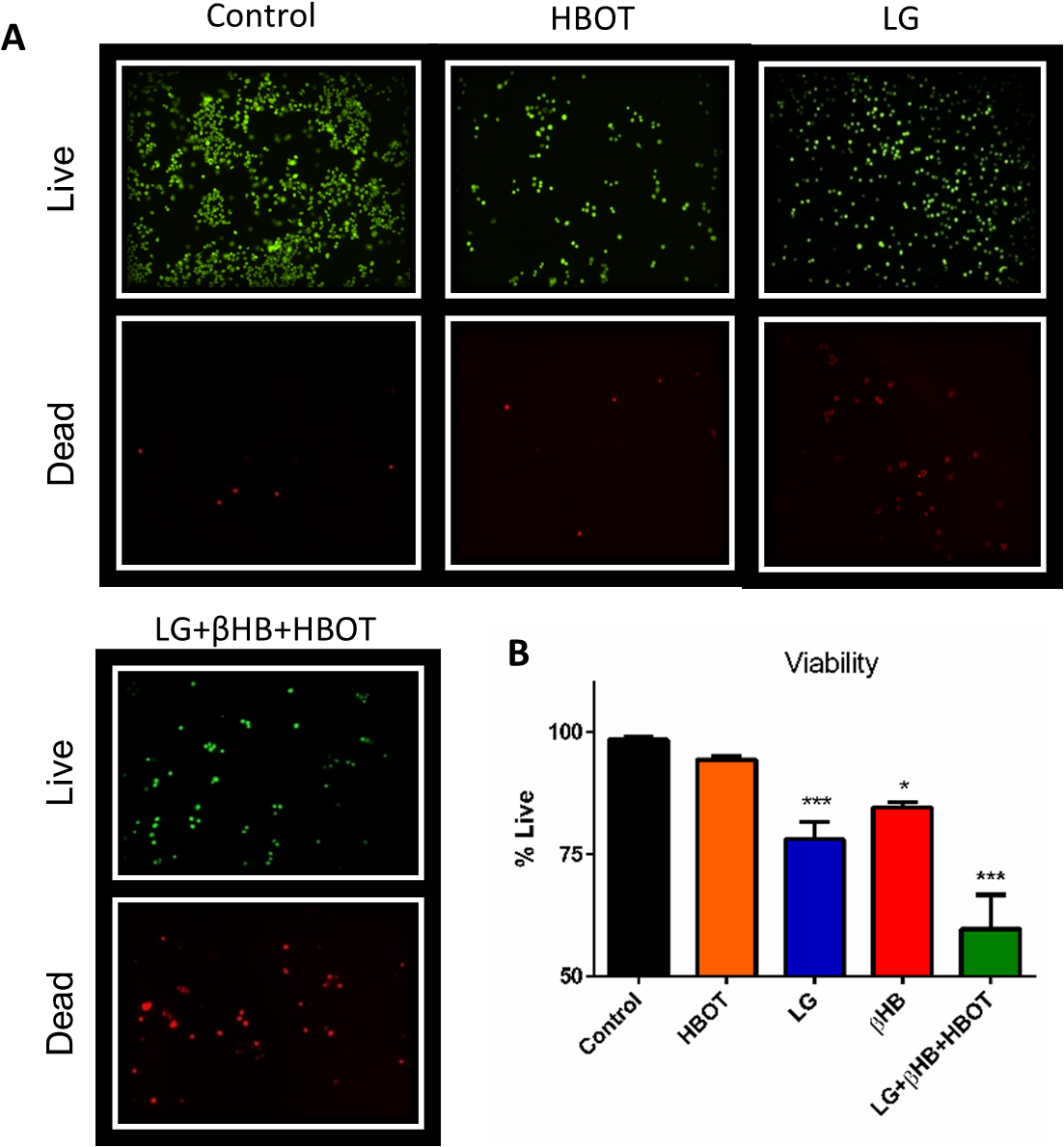
Combination metabolic therapy decreases VM-M3 cell viability *in vitro*. VM-M3 cells were treated with high (control; 25mM) or low (LG; 3mM) glucose for 24 hours, with or without 5mM βHB or one session of HBOT. Viability was measured with calcein AM and Ethd-1 fluorescence microscopy. **(A-B)** βHB, LG, and LG+βHB+HBOT treatment decreased viability compared to control and HBOT treated cells. LG+βHB+HBOT cell viability was significantly decreased compared to βHB but not LG treated cells. Results were considered significant when p<0.05. Error bars represent **±**SEM.

**Fig 8:**
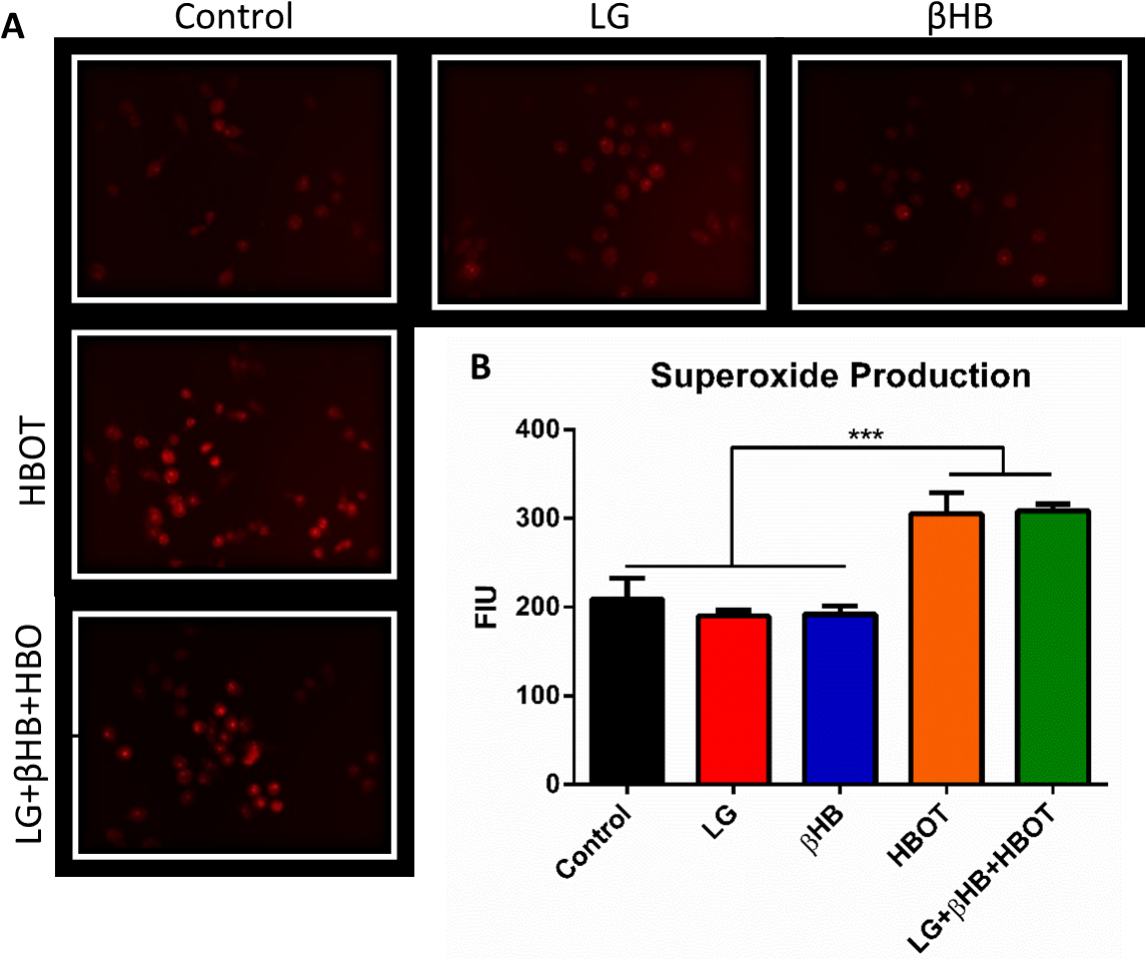
Metabolic therapy increases ROS production in VM-M3 cells. **(A-B)** VM-M3 cells were treated for 24 hours with individual or combination metabolic therapies: LG, βHB, HBOT, or LG+βHB+HBOT. Superoxide production was measured with DHE fluorescence microscopy and was significantly increased with HBOT alone or in combination with LG+βHB. Results were considered significant when p<0.05. Error bars represent **±**SEM.

Proliferation of VM-M3 cells was significantly inhibited by decreasing the glucose concentration of the culture media and by administering HBOT (Fig 6A). We previously reported a similar response in VM-M3 cells with βHB supplementation to varied glucose concentration [22]. Combining LG, βHB, and HBOT elicited potent anti-proliferative effects *in vitro* (Fig 6B). Cells receiving this combination therapy divided very slowly, with a marked significant decrease in cell density compared to controls at all time points tested (*p<0.05, one-way ANOVA; Fig 6B). At 24 and 48 hours, growth rate was significantly slower in the LG+HBOT cells than in the control cells. Growth rate was also significantly slower in the LG+βHB+HBOT cells than in the LG cells. The differences in proliferation rate between the groups became more distinct with time, such that by 72 hours, the proliferation rate of all treatment groups differed significantly from each other with the exception of LG versus LG+HBOT, and at 96 hours, all groups differed with the exception of LG versus LG+HBOT. There was a similar effect of metabolic therapy on VM-M3 cell viability. Viability was 19% less in the LG group than in the control group at 24 hour treatment (*p<0.05, one-way ANOVA; Fig 7). We previously reported a similar effect in VM-M3 cells treated with βHB supplementation [22]. Although HBOT did not decrease viability on its own, when administered with LG and βHB, this combination therapy significantly decreased viability by approximately 38%. (***p<0.001, one-way ANOVA; Fig 7). VM-M3 cell superoxide production was significantly increased with HBOT, both alone and in combination with LG+βHB (***p<0.001, one-way ANOVA; Fig 8).

## Discussion

Metastasis remains the largest obstacle in identifying effective therapies for the long-term management of cancer. A lack of animal models which reflect the metastatic phenotype prevents major improvements in patient care. The major goal of this study was to investigate the anti-cancer efficacy of a novel combination of metabolic therapies—the ketogenic diet, ketone supplementation, and hyperbaric oxygen—in a mouse model of aggressive metastatic cancer that recapitulates much of the metastatic phenotype seen in invasive human cancers [53, 57–59]. Individually, these therapies inhibited proliferation and viability *in vitro*, slowed disease progression *in vivo*, and when combined, elicited potent anti-cancer effects by inhibiting metastatic spread and doubling the survival time of mice with systemic, metastatic disease.

We previously found that ketone supplementation and combination ketogenic diet with HBOT slowed cancer progression in the VM-M3 model of metastatic cancer [9, 22]. We hypothesized that combining these therapeutic regimens could provide an even more significant response in this model. The KD has been used clinically to treat pediatric refractory epilepsy for nearly a century, and is known to be a safe and feasible option for patients [60, 61]. HBOT is an approved therapy for several disease states, including decompression sickness, carbon monoxide poisoning, and radionecrosis [62]. Its use and safety is well-characterized and understood [62, 63]. Exogenous ketone ester supplementation is a novel therapy that is also safe and feasible [64, 65]. When ketone supplementation is administered properly, blood ketone levels remain in normal physiological levels and do not approach the dangerous levels associated with diabetic ketoacidosis (15–20 mM). Ketone supplementation can be used, however, to elevate blood ketone levels to a therapeutic range of 1-5mM [22], which is reported to be beneficial in an array of disease states, including epilepsy, Alzheimer’s disease, and other neurological disorders [60, 66–72]. Importantly, these treatments represent non-toxic alternative or adjuvant therapies, which could be readily implemented clinically if their effects hold up in human trials, especially in cases where standard of care is not advised or has limited efficacy.

The VM-M3 model exhibits rapid, systemic metastasis following s.c. inoculation [53]. As demonstrated by the *in vivo* bioluminescent imaging (Figs 1 and 2), primary tumor growth and pattern of metastatic spread can be highly variable, such as is seen in many human metastatic cancers. Combination KD, ketone supplementation, and HBOT treatment induced a trend of slowed tumor growth rate *in vivo* (Fig 1). Overall tumor burden of these combination therapy animals was significantly decreased compared to control animals at all applicable time points (weeks 1, 2, and 3), and was also significantly decreased compared to KD treated mice at weeks 3 and 5 and KD+KE treated mice at weeks 1, 2, 3, and 5 (Fig 1). Livers of KD+KE+HBOT treated mice exhibited only micrometastatic lesions with little notable vascularization compared to controls at day 21 (Fig 4). These results are consistent with the literature where others have reported an anti-angiogenic effect of HBOT in tumors. Stuhr et. al reported that HBOT decreased vascular density and tumor growth in DMBA-induced rat mammary tumors [50, 73]. Pro-angiogenic genes, including VEGF, FGF, PDGF, and TGF-α were down-regulated in the HBOT-treated tumors. Therefore, our data suggest that KD+KE+HBOT combination therapy may work in part by inhibiting tumor vascularization, which likely contributes to its potent ability to prolong survival time in VM-M3 mice (Fig 4).

Importantly, the data presented in this manuscript can be directly compared to our two previous works investigating the proposed therapies individually (S1 and S2 Files) [9, 22]. The animal studies from these three manuscripts were performed in back-to-back phases, in which a portion of the control animals were implanted during each phase. We had previously reported that combining the KD with HBOT increased survival time in the VM-M3 model by 77.9% (S1 File) and that KE treatment alone increased survival time by 69.2% (S2 File) [9, 22]. Not surprisingly considering the variability in animal models, while mean survival time of animals receiving the multi-combination therapy was increased by 103% compared to controls, it was not significantly different than those receiving any of our individual therapies alone [9]. Nonetheless, due to the strong trends of improved response with multi-combination *in vitro* and *in vivo*, we suggest that the multi-combination of KD+KE+HBOT regimen is a promising method of administering these metabolic therapies. The KD and HBOT have been investigated as potential adjuvant cancer therapies in a number preclinical studies, demonstrating not only efficacy as individual treatments but significant synergy with other adjuvant therapies or standard of care including chemotherapy and radiation [46, 74]. Due to these promising studies, the KD is being investigated in many current or upcoming animal studies and clinical trials. Ketone supplementation is a novel therapy that would likely allow patients to more easily achieve therapeutic levels of ketosis without the need to adhere to as strict a dietary regimen than when using KD alone. Indeed, preliminary studies in our lab indicate that chronic consumption of ketone ester induces sustained therapeutic ketosis in healthy rats consuming a carbohydrate-based diet [75].

The effect of these metabolic therapies on proliferation and viability *in vitro* mimicked the effects seen *in vivo*. Both βHB and HBOT significantly decreased VM-M3 cell proliferation, and this effect was enhanced by decreasing blood glucose (Fig 3) [22]. We showed previously that βHB decreased VM-M3 cell viability, even in the presence of high glucose, but this effect was enhanced by lowering glucose concentration [22]. HBOT alone did not increase survival time *in vivo*, and although there was a trend of decreased viability *in vitro*, this was not statistically significant; however, combining HBOT with ketosis elicited potent anti-cancer effects in mice and cells (Fig 2) [9]. Taken together, these studies suggest that combining therapeutic ketosis with HBOT creates a unique physiologic and metabolic environment that is not conducive to rapid cancer cell proliferation and survival.

Blood glucose and body weight decreased and blood βHB increased in mice receiving KD+KE therapy by day 7, prior to the onset of significant disease progression (Fig 5). As would be expected, blood glucose was lower and blood βHB was elevated in KD+KE mice compared to KD alone. KD+KE mice lost approximately 20% of their initial body weight, suggesting a potential therapeutic contribution from calorie restriction. However, we previously showed that the anti-cancer effects of KE supplementation was not due to CR in body weight matched animals [22]. Interestingly, at day 7, KD+KE+HBOT treated mice had increased blood βHB, but blood glucose and body weight were not significantly different from controls. The discrepancy between these two groups receiving the same dietary therapy is unclear. Alteration of tissue oxygenation is known to affect metabolic capacity and blood metabolite levels [76]. Blood glucose decreases immediately following HBOT sessions in both animals and humans [77, 78]. This may be due to an increase in insulin secretion and tissue glucose utilization which has been reported with HBOT [79–82]. In our study, blood metabolites were measured several hours after HBOT. Perhaps HBOT-induced glucose utilization by the brain and peripheral tissues stimulated gluconeogenesis or feeding behavior in the VM-M3 mice. This could possibly explain why glucose was not decreased at the time of blood measurement and why these animals did not lose weight as the KD+KE mice did. Similarly, HBOT has been shown to enhance weight gain in head and neck cancer patients following surgery and radiation [83]. It is possible that, as in our combination treatment group, patients using HBOT would find it more difficult to reduce blood glucose to therapeutic levels. Therefore, it would be important for individuals implementing these therapeutic options to closely monitor blood glucose and ketone levels and adjust their dietary habits to maintain a state of nutritional ketosis which we propose is important for maximal therapeutic benefit. These changes would likely include increasing carbohydrate restriction or introducing caloric restriction or intermittent fasting, all of which have been reported to have anti-cancer effects on their own [74].

As expected, HBOT increased ROS production in VM-M3 cells when administered alone, or in combination with LG+βHB (Fig 8). Although cancer cells thrive in a state of elevated basal ROS production, they are very sensitive to modest increases or decreases in ROS or alterations in redox or antioxidant state [84, 85]. Furthermore, the differential susceptibility of cancer and normal cells to glucose deprivation has been shown to be mediated by superoxide and hydrogen peroxide production and signaling [86, 87]. This may help explain why combining HBOT with glucose restriction, such as with the KD, elicit synergistic effects *in vivo* [9]. Combining ketosis with hyperbaric oxygen may specifically target cancer metabolism while supporting the health of normal tissues and preventing the many negative symptoms of disease and standard care, like metabolic syndrome and radiation necrosis [88, 89]. We and others have shown that many cancers are unable to effectively metabolize ketones; however, they are an efficient and protective energy source for healthy tissues [22, 25, 72]. Ketone metabolism decreases ROS production and increases the endogenous antioxidant profile of cells which consume them [72, 90]. Due to the abnormal mitochondrial function present in most cancers, it is unclear if ketone metabolism would reduce oxidative stress in tumors as it does in healthy tissue. While some studies have reported the ability of ketones to reduce ROS production in cancer models [13, 91], other studies have reported enhanced oxidative stress in tumors of animals treated with the KD [92], and in our current study, there was no change in ROS production with ketone treatment (Fig 8). This phenomenon will be important to understand as we further explore the use of the KD and ketone supplementation in cancer; however, it has not been well-studied to date. The discrepancies between the reported data are likely due to differences between cancer types and the degree to which mitochondria are dysfunctional in each particular cancer. Since many therapies, including chemotherapy, radiation, and HBOT, work in part by increasing ROS production in the tumor, simultaneous ketone metabolism by surrounding healthy tissue may work to prevent toxic side effects in these tissues. Both ketosis and HBOT have been shown to sensitize tumors to radiation and chemotherapy [20, 46, 93, 94]. Concurrent administration of these therapies may allow for the delivery of standard care at reduced doses, lessening toxic side effects and supporting the health of the individual. Importantly, βHB did not decrease ROS production in the VM-M3 cells (Fig 8), further supporting the notion that these cancer cells are not able to metabolize ketones as healthy cells do.

There is understandable concern regarding the manipulation of diet in vulnerable cancer patients who may find it difficult to consume adequate calories [95]. If these adjuvant therapies allow for decreased yet effective doses of chemotherapy and radiation, patients will likely have an easier time consuming enough calories to support their metabolic needs. Indeed, late-stage patients given the KD report improved quality of life, and small clinical studies suggest that many patients are willing and able to implement the dietary restrictions of the ketogenic diet [11, 17, 20, 96]. Simultaneous administration of a ketone supplement such as the ketone ester could potentially provide the same benefit of a KD without the need for such restrictive food intake [22]. Furthermore, ketone metabolism is a protein sparing process [97]. The human brain evolved to metabolize ketones as a way to spare muscle protein during prolonged periods of food deprivation [71]. Ketogenic diets promote weight loss in overweight patients, but they are also known to prevent muscle wasting during physical stress, energy restriction, or starvation [98–101]. In a small study on humans with advanced cancer and cachexia, patients maintained a positive nitrogen balance and gained weight when given a ketone-supplemented ketogenic diet [102]. Indeed, an elevation in blood ketones decreases amino acid release from muscle and decreases hepatic gluconeogenesis of amino acids [72]. Similarly, mice given a KD had significantly smaller tumors but did not lose as much body weight as control animals in a murine model of cancer cachexia [15]. Furthermore, control animals had high levels of circulating catabolic factors which elicit triglyceride and amino acid release, but this was inhibited by βHB [15, 102]. Similar studies showed that in mice with a colon cancer that elicited a severe cachexic phenotype, the brain greatly increased expression of 3-oxoacid CoA-transferase, a ketone utilizing enzyme, and relied more heavily on βHB for energy [103]. Enhancing ketone availability as an alternative fuel to the brain and other healthy tissues with the ketogenic diet and/or ketone supplementation will likely support their function during the cachexic state.

There is potential for synergy between these adjuvant metabolic therapies and standard care [52, 54, 96, 104]. Scheck and colleagues reported a significant synergistic effect of combining the KD with radiation therapy in a mouse model of brain cancer [93]. Other studies have demonstrated increased efficacy by pairing the KD with chemotherapy or pharmacological agents which target metabolism [20, 94]. HBOT has been investigated as a radiosensitizer in a number of clinical studies [52, 105]. Meta-analyses conclude that HBOT appears to have a neutral or inhibiting effect on cancer progression, depending on cancer type [46, 51, 52]. When administered concurrently with radiation, HBOT potentiates efficacy, but may also increase soft tissue necrosis in nearby healthy tissue [46]. Studies indicate that this adverse effect can be mitigated by delivering HBOT immediately prior to, rather than concurrent with, radiation therapy [44]. Indeed, in this study, human glioma patients receiving HBOT immediately prior to radiation therapy had a median survival time of 24 months, compared to 12 months in patients receiving radiation alone, and did not experience significant side effects [44]. As previously discussed, we hypothesize that ketone metabolism by the healthy tissue may also mitigate adverse effects and perhaps potentiate the efficacy of HBOT and/or standard care. We previously showed that HBOT alone did not affect survival in the VM-M3 model, but dramatically slowed progression when administered with a KD [9]. Perhaps other studies that found no effect of HBOT could have revealed therapeutic potential by coupling this therapy with ketosis, either by the KD or ketone supplementation.

Despite 50 years of intensive research, cancer remains the second leading cause of death in the United States. This is largely due to a lack of therapies effective for long-term management of metastatic disease, a dilemma underlined by inadequate preclinical models. In this study, we evaluated the efficacy of a novel, non-toxic combination metabolic therapy—the ketogenic diet, ketone supplementation, and hyperbaric oxygen—in a syngeneic mouse model of metastatic disease which we believe reliably reflects the human metastatic phenotype [53, 57–59]. Our study suggests that this combination therapy presents a safe, cost-effective, adjuvant to standard care which could provide novel therapeutic options for patients with late-stage cancer. We believe that it is critically important to evaluate non-toxic adjuvant therapies like these, and to look for synergistic potential with other metabolic therapies and standard care. It is possible that we already possess therapeutic options which could significantly improve patient prognosis or outcome when delivered in the appropriate combinations.

## Supporting Information

S1 File Combination ketogenic diet and hyperbaric oxygen as a novel cancer therapy—Related Manuscript. This related work was published in PLOS One in 2013 and is a relevant and direct companion to this study.
(PDF)

S2 File Exogenous ketone supplementation as a novel cancer therapy—Related Manuscript. This related work was published in the International Journal of Cancer in 2013 and is a relevant and direct companion to this study.
(PDF)

S3 File Study data. (XLSX)
