# Supplementary material for: Non-Toxic Metabolic Management of Metastatic Cancer in VM Mice: Novel Combination of Ketogenic Diet, Ketone Supplementation, and Hyperbaric Oxygen Therapy
Source: PLoS One. 2015 Jun 10;10(6):e0127407. doi: 10.1371/journal.pone.0127407 (PMC4464523; doi:10.1371/journal.pone.0127407)
Supplement: S1 File — This related work was published in PLOS One in 2013 and is a relevant and direct companion to this study. (PDF) [file pone.0127407.s001.pdf]

OPEN ACCESS Freely available online

PLOS ONE

The Ketogenic Diet and Hyperbaric Oxygen Therapy Prolong Survival in Mice with Systemic Metastatic Cancer

Angela M. Poff[1]*, Csilla Ari[1], Thomas N. Seyfried[2], Dominic P. D'Agostino[1]

1 Department of Molecular Pharmacology and Physiology, University of South Florida, Tampa, Florida, United States of America, 2 Department of Biology, Boston College, Chestnut Hill, Massachusetts, United States of America

Abstract

Introduction: Abnormal cancer metabolism creates a glycolytic-dependency which can be exploited by lowering glucose availability to the tumor. The ketogenic diet (KD) is a low carbohydrate, high fat diet which decreases blood glucose and elevates blood ketones and has been shown to slow cancer progression in animals and humans. Abnormal tumor vasculature creates hypoxic pockets which promote cancer progression and further increase the glycolytic-dependency of cancers. Hyperbaric oxygen therapy (HBO_2T) saturates tumors with oxygen, reversing the cancer promoting effects of tumor hypoxia. Since these non-toxic therapies exploit overlapping metabolic deficiencies of cancer, we tested their combined effects on cancer progression in a natural model of metastatic disease.

Methods: We used the firefly luciferase-tagged VM-M3 mouse model of metastatic cancer to compare tumor progression and survival in mice fed standard or KD *ad libitum* with or without HBO_2T (2.5 ATM absolute, 90 min, 3x/week). Tumor growth was monitored by *in vivo* bioluminescent imaging.

Results: KD alone significantly decreased blood glucose, slowed tumor growth, and increased mean survival time by 56.7% in mice with systemic metastatic cancer. While HBO_2T alone did not influence cancer progression, combining the KD with HBO_2T elicited a significant decrease in blood glucose, tumor growth rate, and 77.9% increase in mean survival time compared to controls.

Conclusions: KD and HBO_2T produce significant anti-cancer effects when combined in a natural model of systemic metastatic cancer. Our evidence suggests that these therapies should be further investigated as potential non-toxic treatments or adjuvant therapies to standard care for patients with systemic metastatic disease.

Citation: Poff AM, Ari C, Seyfried TN, D'Agostino DP (2013) The Ketogenic Diet and Hyperbaric Oxygen Therapy Prolong Survival in Mice with Systemic Metastatic Cancer. PLoS ONE 8(6): e65522. doi:10.1371/journal.pone.0065522

Editor: Chih-Hsin Tang, China Medical University, Taiwan

Received December 20, 2012; **Accepted** May 2, 2013; **Published** June 5, 2013

Copyright: © 2013 Poff et al. This is an open-access article distributed under the terms of the Creative Commons Attribution License, which permits unrestricted use, distribution, and reproduction in any medium, provided the original author and source are credited.

Funding: This work was supported by the Office of Naval Research, ONR grant N000140610105 and ONR-DURIP equipment grant N000140210643 (http://www.onr.navy.mil/). The funders had no role in study design, data collection and analysis, decision to publish, or preparation of the manuscript.

Competing Interests: The authors have declared that no competing interests exist.

* E-mail: abennett@health.usf.edu

Introduction

Metastasis is a complex phenomenon in which cancer cells spread from a primary tumor to establish foci in a distal tissue and is responsible for 90 percent of cancer-related deaths [1]. The specific changes which mediate metastasis remain unclear; however, the process generally involves local tumor growth, invasion through the basement membrane and surrounding tissue, intravasation into the lymphatics or blood vessels, dissemination and survival in circulation, extravasation from the vasculature, and re-establishment of tumors at distal tissues. While many primary tumors can be controlled with conventional therapies like surgery, chemotherapy, and radiation, these treatments are often ineffective against metastatic disease and in some cases may promote cancer progression and metastasis [2,3,4]. There is a substantial need for novel therapies effective against metastatic cancer.

Perhaps the most important limiting factor in the development of new treatments for metastatic cancer is the lack of animal models that accurately reflect the true nature of metastatic disease. Xenograft models of human cancers in immunodeficient mice are inadequate as the immune system is highly involved in cancer development and progression. Indeed, most tumor models grown as xenografts in immune compromised mice fail to metastasize [5,6]. Tail vein injection models of metastatic cancer eliminate the important steps of local tissue invasion and intravasation into the vasculature, again failing to represent the true disease phenotype. The VM-M3 model of metastatic cancer is a novel murine model that closely mimics the natural progression of invasion and metastasis [7,8]. The VM-M3 tumor arose spontaneously in the brain of a mouse of the VM/Dk inbred strain and expresses multiple growth characteristics of human glioblastoma multiforme with macrophage/microglial properties [7,9]. When implanted subcutaneously, VM-M3 cells rapidly metastasize to all major organ systems, notably the liver, lung, kidney, spleen, brain, and bone marrow. Systemic metastasis has also been repeatedly documented in human glioblastoma multiforme (GBM), which has been linked to the macrophage/microglial characteristics of the tumor [9]. The tumor was adapted to cell culture and transfected with the firefly luciferase gene to allow for easy monitoring of tumor growth *in vivo* [10]. The VM-M3 model of

metastatic cancer has a distinct advantage over other metastatic models because it spreads naturally in an immunocompetent host, mimicking the natural cancer microenvironment. Cisplatin and methotrexate, two commonly-used chemotherapy agents, inhibit metastatic spread in the VM-M3 model of metastatic cancer similarly to their effects in humans, further supporting the model's representation of the true disease state [8,11,12,13]. For these reasons, the VM-M3 model of metastatic cancer was used for this study.

Abnormal energy metabolism is a consistent feature of most tumor cells across all tissue types [14]. In the 1930 s, Otto Warburg observed that all cancers expressed high rates of fermentation in the presence of oxygen [15]. This feature, known as The Warburg Effect, is linked to mitochondrial dysfunction and genetic mutations within the cancer cell [14,16,17]. These defects cause cancers to rely heavily on glucose for energy, a quality that underlies the use of fluorodeoxyglucose-PET scans as an important diagnostic tool for oncologists [18]. Ketogenic diets are high fat, low carbohydrate diets that have been used for decades to treat patients with refractory epilepsy [19]. Ketogenic diets also suppress appetite naturally thus producing some body weight loss [19,20,21,22]. Dietary energy reduction (DER) lowers blood glucose levels, limiting the energy supply to cancer cells, while elevating circulating blood ketone levels [6]. Ketone bodies can serve as an alternative energy source for those cells with normal mitochondrial function [23,24], but not for cancer cells [25]. DER has been shown to have anti-tumor effects in a variety of cancers, including brain, prostate, mammary, pancreas, lung, gastric, and colon [14,26,27,28,29,30,31,32,33,34]. DER produces anti-cancer effects through several metabolic pathways, including inhibition of the IGF-1/PI3K/Akt/HIF-1α pathway which is used by cancer cells to promote proliferation and angiogenesis and inhibit apoptosis [35,36,37,38,39,40,41,42]. Additionally, DER induces apoptosis in astrocytoma cells, while protecting normal brain cells from death through activation of adenosine monophosphate kinase (AMPK) [43].

Tumors possess abnormal vasculature which blocks adequate tissue perfusion, leading to the presence of hypoxic regions that promote chemotherapy and radiation resistance [44,45,46,47]. In fact, hypoxic cancer cells are three-times more resistant to radiation therapy than are well-oxygenated cells [48]. In addition to decreasing the efficacy of standard care, tumor hypoxia activates a number of oncogene pathways, largely through the HIF-1 transcription factor, which promote tumor growth, metastasis, angiogenesis, and inhibit apoptosis [49,50].

Hyperbaric oxygen therapy (HBO_2T) involves administration of 100% oxygen at elevated pressure (greater than sea level, or 1 ATA). HBO_2T increases plasma oxygen saturation which facilitates oxygen delivery to the tissue independent of hemoglobin O_2 saturation [51]. The potential benefit of using HBO_2T to combat the cancer-promoting effects of tumor hypoxia is clear. HBO_2T alone has been shown to inhibit tumor growth, reduce tumor blood vessel density, and induce the preferential expression of anti-cancer genes in rat models of mammary tumors [52]. Additionally, radiation and many chemotherapy drugs work by producing free radicals within the tumors, leading to cell death. HBO_2T enhances tumor-cell production of reactive oxygen species which contributes to the synergistic effects of HBO_2T as an adjuvant treatment to standard care. Indeed, HBO_2T enhances the efficacy of both radiation and chemotherapy in animal models [53,54,55,56,57].

In normal tissues, decreased oxygen availability inhibits mitochondrial production of ATP, stimulating an up-regulation of glycolytic enzymes to meet energy needs by substrate level phosphorylation production of ATP. Thus, the cellular response to tumor hypoxia is mediated by several of the same pathways that are overly active in cancer cells with mitochondrial damage and high rates of aerobic glycolysis. This suggests that the ketogenic diet and HBO_2T could target several overlapping pathways and tumorigenic behaviors of cancer cells. We hypothesized that these treatments would work synergistically to inhibit tumor progression. We suggest that the addition of these non-toxic adjuvant therapies to the current standard of care may improve progression free survival in patients with advanced metastatic disease.

Materials and Methods

Mice

Three breeding pairs of the VM/Dk strain of mice were used to establish and propagate a VM/Dk mouse colony in the University of South Florida (USF) Morsani College of Medicine Vivarium according to standard husbandry protocol. Forty adult male mice (10–18 weeks of age) were used for this study. All animal procedures were performed within strict adherence to the NIH Guide for the Care and Use of Laboratory and Animals and were approved by the USF Institutional Animal Care and Use Committee (IACUC; Protocol Number R4137).

Cell Culture

VM-M3/Fluc cells were received as a gift from T.N. Seyfried, Boston College, where they were created from a spontaneous tumor in a VM/Dk mouse and adapted to cell culture [7]. VM-M3/Fluc cells were transduced with a lentivirus vector containing the firefly luciferase gene under control of the cytomegalovirus promoter (VM-M3/Fluc) as previously described [7]. The VM-M3/Fluc cells were cultured in Eagle's Minimum Essential Medium with 2 mM L-glutamine (ATCC, Manassas, VA), 10% fetal bovine serum (Invitrogen, Grand Island, NY), 1% penicillin-streptomycin (Gibco, Invitrogen) and high glucose (25 mM D-glucose, Fisher Scientific, Waltham, MA). Cells were cultured in a CO_2 incubator at 37°C in 95% air and 5% CO_2.

Subcutaneous Tumor Implantation

On day 0, VM-M3/Fluc cells (1 million cells in 300 µL PBS) were implanted, s.c., into the abdomen of VM/Dk mice using a 27 gage needle. Inoculation results in rapid and systemic metastasis to most major organs, namely liver, kidneys, spleen, lungs, and brain as previously described [7].

Diet Therapy

On the day of tumor inoculation, mice were randomly assigned to one of four groups: SD (Control); SD+HBO_2T; KD; or KD+HBO_2T. Mice in the SD group were fed standard rodent chow (2018 Teklad Global 18% Protein Rodent Diet, Harlan) *ad libitum.* Mice in the KD group received KD-Solace ketogenic diet *ad libitum*. KD-Solace is a commercially available ketogenic diet powder (KetoGen, Solace Nutrition) and was mixed 1:1 with H_2O to form a solid paste. Macronutrient information for SD and KD-Solace are shown in Table 1. Diets were continuously replaced every other day to maintain freshness and allow mice to feed *ad libitum.*

Hyperbaric Oxygen Therapy (HBO_2T)

Mice undergoing HBO_2T received 100% O_2 for 90 minutes at 1.5 ATM gauge (2.5 ATM absolute) three times per week (M, W, F) in a hyperbaric chamber (Model 1300B, Sechrist Industries, Anaheim, CA).

Table 1. Macronutrient information of diets.

Macronutrient Information	Standard Diet	KD-Solace
% Cal from Fat	18.0	89.2
% Cal from Protein	24.0	8.7
% Cal from Carbohydrate	58.0	2.1
Caloric Density	3.1 Kcal/g	7.12 Kcal/g

doi:10.1371/journal.pone.0065522.t001

Glucose, Ketone, and Weight Measurements

Every 7 days, blood was collected from the tail using approved methods. Glucose was measured using the Nova Max® Plus™ Glucose and β-Ketone Monitoring System (Nova Biomedical, Waltham, MA), and β-hydroxybutyrate was measured using the Precision Xtra™ Blood Glucose & Ketone Monitoring System (Abbott Laboratories, Abbott Park, IL).

Mice were weighed between 1 and 3 pm twice a week for the duration of the study using the AWS-1KG Portable Digital Scale (AWS, Charleston, SC).

Bioluminescent Imaging and Tumor Growth Analysis

Tumor growth was monitored as a measure of bioluminescent signaling using the Xenogen IVIS Lumina system (Caliper LS, Hopkinton, MA). Data acquisition and analysis was performed using the Living Image® software (Caliper LS). Approximately 15 minutes prior to *in vivo* imaging, the mice received an i.p. injection of D-Luciferin (50 mg/kg) (Caliper LS). Bioluminescent signal was obtained using the IVIS Lumina cooled CCD camera system with a 1 sec exposure time. As only the cancer cells contained the luciferase gene, bioluminescent signal (photons/sec) of the whole animal was measured and tracked over time as an indicator of metastatic tumor size and spread.

Survival Analysis

Throughout the study, health and behavior of the mice were assessed daily. Mice were humanely euthanized by CO_2 asphyxiation according to IACUC guidelines upon presentation of defined criteria (tumor-associated ascites, diminished response to stimuli, lethargy, and failure to thrive), and survival time was recorded.

Statistics

Survival data was analyzed by the Kaplan-Meier and Logrank Tests for survival distribution. Mean survival times were analyzed by two-tailed student's t-tests. Bioluminescent signal as a measure of tumor size was analyzed by two-tailed student's t-tests. Blood measurements were analyzed by One Way ANOVA with Kruskal Wallis Test and Dunn's Multiple Comparison Test post hoc. Differences in percent weight change were analyzed by One Way ANOVA with Tukey's Multiple Comparison Test post hoc. Correlation between blood glucose, body weight change, and survival times were analyzed by linear regression analysis. Results were considered significant when $p<0.05$.

Results

Combining the KD with HBO_2T Prolonged Survival in Mice with Metastatic Cancer

KD and KD+HBO_2T treated mice demonstrated a statistically different survival curve by Logrank Test with an increase in survival time compared to control animals ($p = 0.0194$ and $p = 0.0035$, respectively; Figure 1A). KD fed and KD+HBO_2T animals also showed a significant increase in mean survival time compared to control animals by the two-tailed student's t-test ($p = 0.0044$ and $p = 0.0050$, respectively; Figure 1B). Although previous studies have reported that HBO_2T alone can increase survival time in animals with various cancers [52,54,58,59], we did not see an effect on survival in mice receiving SD+HBO_2T. Control (SD) mice had a mean survival time of 31.2 days whereas SD+HBO_2T mice had a non-statistically different mean survival of 38.8 days (Figure 1B). The KD alone increased mean survival time by approximately 17 days (56.7%), and when combined with HBO_2T, mice exhibited an increase in mean survival time of approximately 24 days (77.9%) (Figure 1B). This finding strongly supports the efficacy of the KD and HBO_2T as therapies to inhibit tumor progression and prolong survival in animals with metastatic cancer.

The KD and HBO_2T Decreased Tumor Bioluminescence

Bioluminescent signal was tracked as a measure of tumor size throughout the study. Animals receiving the KD alone or in combination with HBO_2T demonstrated a notable trend of slower tumor growth over time. This trend was more pronounced in KD+HBO_2T mice and reflected the increase in survival time seen in these animals (Figures 1, 2). The difference in mean tumor size between KD+HBO_2T and control animals at week 3 was statistically significant ($p = 0.0062$; Figure 2B). Day 21 *ex vivo* organ bioluminescence of KD+HBO_2T mice demonstrated a trend of reduced metastatic tumors in animals compared to the SD group (Figure 2). Spleen bioluminescence was significantly decreased in KD+HBO_2T mice ($p = 0.0266$).

The KD Lowered Blood Glucose, Elevated Blood Ketones, and Decreased Body Weight

Prior to the study, initial blood glucose, ketone, and body weights were similar among the groups (data not shown). Blood glucose levels were lower in the KD-treated mice than in the SD-treated mice by day 7 ($p<0.001$; Figure 3). While all KD-fed mice demonstrated a trend of elevated blood ketone levels throughout the duration of the study, only the KD+HBO_2T animals showed significantly increased ketones compared to controls on day 7 ($p<0.001$; Figure 3). By day 7, KD-fed mice lost approximately 10% of their initial body weight and maintained that weight for the duration of the study (Figure 4). Day 7 blood glucose and percent body weight change were significantly correlated to survival time ($p = 0.0189$ and $p = 0.0001$, respectively; Figure 5).

Discussion

Nearly a century after Otto Warburg reported the abnormal energy metabolism of cancer cells, renewed interest in the field has elucidated a plethora of novel therapeutic targets. Two promising treatments involve the use of HBO_2T to reverse the cancer-promoting effects of tumor hypoxia and the use of the KD to limit the availability of glycolytic substrates to glucose-addicted cancer cells. Both therapies have been previously reported to possess anti-cancer effects [14,54,58,60]. Since these treatments are believed to work by targeting several overlapping mechanisms, we hypothesized that combining these non-toxic treatments would provide a powerful, synergistic anti-cancer effect. Furthermore, since metastasis is responsible for the overwhelming majority of cancer-related deaths, we tested the efficacy of these conjoined therapies on the VM-M3 mouse model of metastatic cancer [7,10].

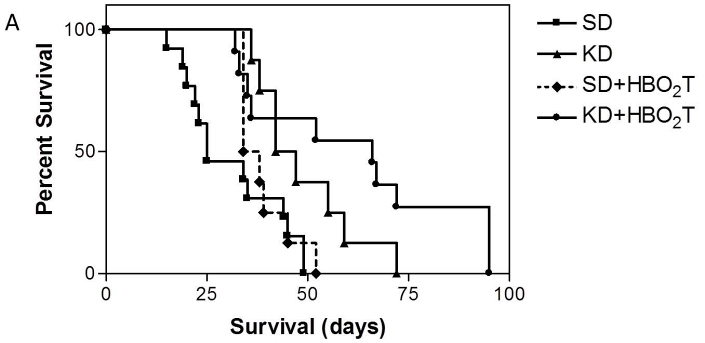

B

Treatment	Cohort Size (N)	Mean Survival Time (days)
**Control (SD)**	13	31.2
**KD**	8	48.9
**SD+HBO_2T**	8	38.8
**KD+HBO_2T**	11	55.5

Figure 1. The KD and HBO_2T increases survival time in mice with systemic metastatic cancer. (**A**) Kaplan-Meier survival plot of study groups. Animals receiving KD and KD+HBO_2T showed significantly longer survival compared to control animals ($p = 0.0194$ and $p = 0.0035$, respectively; Kaplan-Meier and LogRank Tests for survival distribution). (**B**) Treatment group cohort size and mean survival times shown. KD mice exhibited a 56.7% increase in mean survival time compared to controls ($p = 0.0044$; two-tailed student's t-test); KD+HBO_2T mice exhibited a 77.9% increase in mean survival time compared to controls ($p = 0.0050$; two-tailed student's t-test). Results were considered significant when $p<0.05$.
doi:10.1371/journal.pone.0065522.g001

We found that the KD fed *ad libitum* significantly increased mean survival time in mice with metastatic cancer ($p = 0.0194$; Figure 1). It is important to note that KD-fed animals lost approximately 10% of their body weight over the course of the study (Figure 4). It is well established that low carbohydrate, high fat ketogenic diets can cause body weight loss in overweight humans [21,22,61]. Ketogenic diets are also known to have an appetite suppressing effect which may contribute to body weight loss [20]. Along with appetite suppression, a potential contributing factor to the observed body weight loss is the possibility that mice found the KD to be less palatable and were self-restricting caloric intake. As calorie restriction is known to elicit profound anti-cancer effects, the ketogenic diet may inhibit cancer progression in part by indirect dietary energy restriction [6,38]. Fine and colleagues recently used a very low carbohydrate KD to promote stable disease or partial remission in patients with advanced metastatic cancer [62]. Fine's study demonstrated a correlation between blood ketones and response to the diet therapy, suggesting that ketone elevation itself also contributes to the anti-cancer efficacy of the KD.

As hypothesized, profound anti-cancer effects were observed in our metastatic mouse model after combining the KD with HBO_2T. Combining these therapies nearly doubled survival time in mice with metastatic cancer, increasing mean survival time by

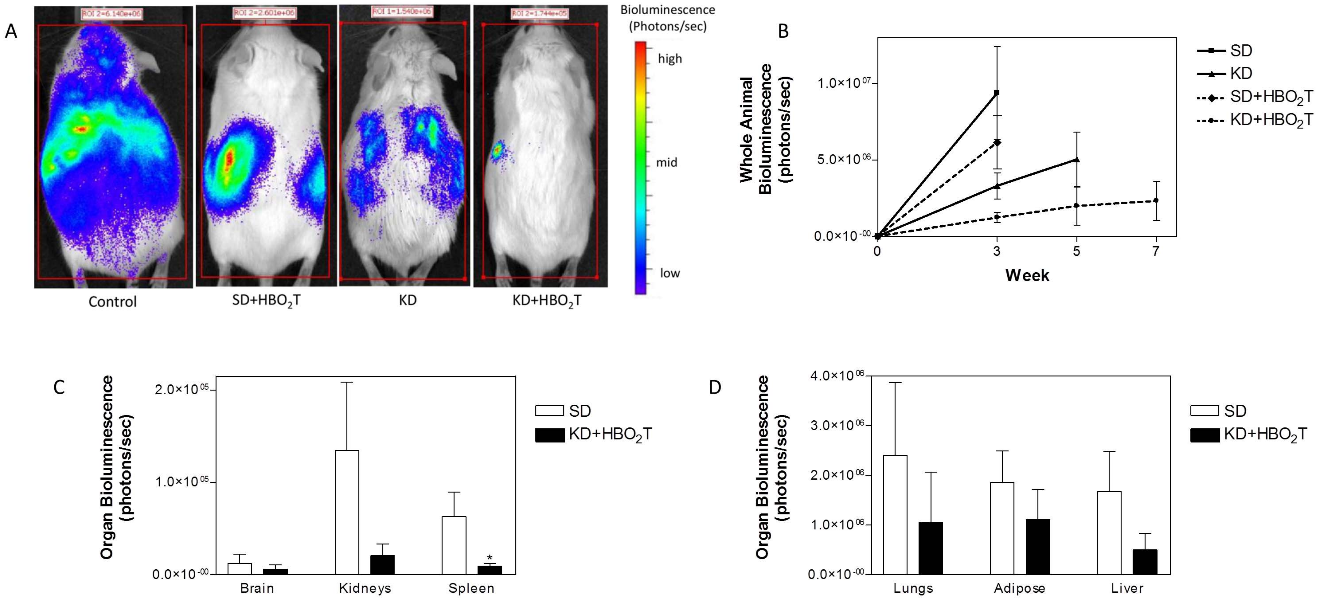

Figure 2. Tumor bioluminescence in mice. Tumor growth was slower in mice fed the KD than in mice fed the SD. (A) Representative animals from each treatment group demonstrating tumor bioluminescence at day 21 after tumor cell inoculation. Treated animals showed less bioluminescence than controls with KD+HBO2T mice exhibiting a profound decrease in tumor bioluminescence compared to all groups. (B) Total body bioluminescence was measured weekly as a measure of tumor size; error bars represent ±SEM. KD+HBO2T mice exhibited significantly less tumor bioluminescence than control animals at week 3 ($p = 0.0062$; two-tailed student's t-test) and an overall trend of notably slower tumor growth than controls and other treated animals throughout the study. (C,D) Day 21 ex vivo organ bioluminescence of SD and KD+HBO2T animals ($N = 8$) demonstrated a trend of reduced metastatic tumor burden in animals receiving the combined therapy. Spleen bioluminescence was significantly decreased in KD+HBO2T mice (*$p = 0.0266$; two-tailed student's t-test). Results were considered significant when $p<0.05$.
doi:10.1371/journal.pone.0065522.g002

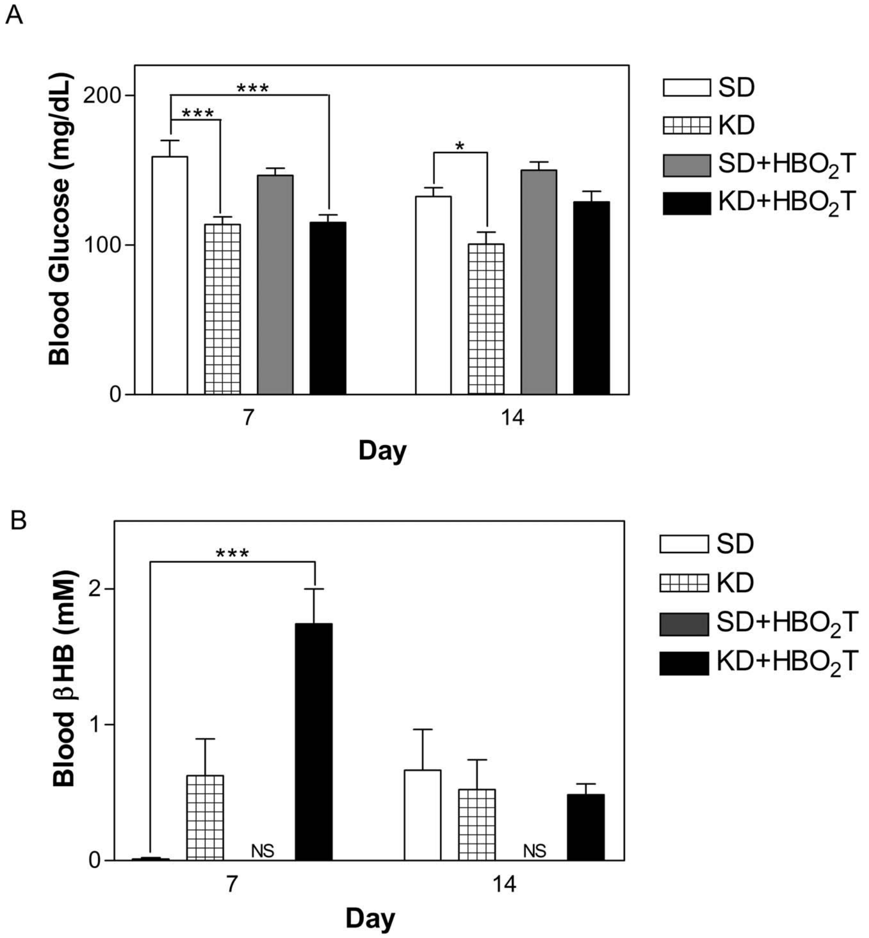

Figure 3. Blood glucose and β-hydroxybutyrate levels in animals. (A) KD-fed mice showed lower blood glucose than controls on day 7 (***$p<0.001$). Animals in the KD study group had significantly lower blood glucose levels than controls on day 14 (*$p<0.05$). (B) KD+HBO2T mice had significantly higher blood ketones than controls on day 7 (***$p<0.001$). Error bars represent ±SEM. Blood analysis was performed with One Way ANOVA with Kruksal Wallis Test and Dunn's Multiple Comparison Test post hoc; results were considered significant when $p<0.05$.
doi:10.1371/journal.pone.0065522.g003

24 days compared to control animals ($p=0.0050$; Figure 1). The KD+HBO_2T-treated mice exhibited significantly decreased bioluminescence compared to controls at week 3 ($p=0.0062$) and a trend of decreased tumor growth rate throughout the study (Figure 2). By day 7, all animals on a ketogenic diet had significantly lower blood glucose levels than controls (Figure 3). As it has been shown that tumor growth is directly correlated to blood glucose levels [63], this decrease in blood glucose concentration likely contributed to the trend of decreased tumor bioluminescence and rate of tumor growth seen in KD-fed animals (Figure 2). Nebeling et al. demonstrated that the KD significantly decreased glucose uptake in pediatric brain tumor patients by FDG-PET analysis [64]. This clinical data suggests decreased glucose delivery to the tumor is a causal mechanism in KD treatment. All KD-fed mice showed a trend of elevated blood ketones throughout the study; however, only KD+HBO_2T mice had significantly higher ketones than controls on day 7 (Figure 3). As ketones are metabolized exclusively within the mitochondria, cancer cells with damaged mitochondria are unable to adequately use them for energy. Many cancers do not express the Succinyl-CoA: 3-ketoacid CoA-Transferase (SCOT) enzyme which is required for ketone body metabolism [65,66]. In fact, βHB administration prevents healthy hippocampal neurons but not glioma cells from glucose withdrawal-induced cell death [24]. Furthermore, ketone bodies have anti-cancer effects themselves, possibly through inhibition of glycolytic enzymes [67]. Skinner and colleagues demonstrated that acetoacetate and βHB administration inhibits brain cancer cell viability *in vitro* [25]. Thus, the elevated ketone

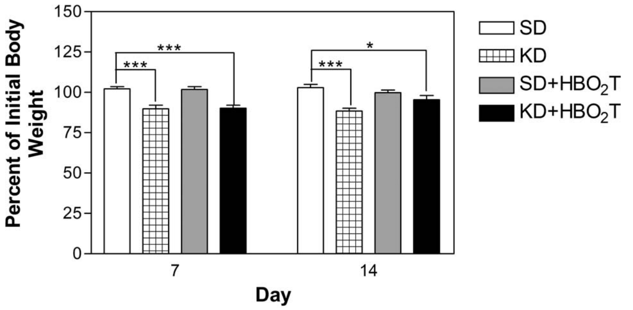

Figure 4. Animal weight. Body weight was measured twice a week. Graph indicates average percent of initial body weight animals at days 7 and 14. KD and KD+HBO2T mice lost approximately 10% of their body weight by day 7 and exhibited a significant difference in percent body weight change compared to control animals (*$p<0.05$; ***$p<0.001$). Error bars represent ±SEM.
doi:10.1371/journal.pone.0065522.g004

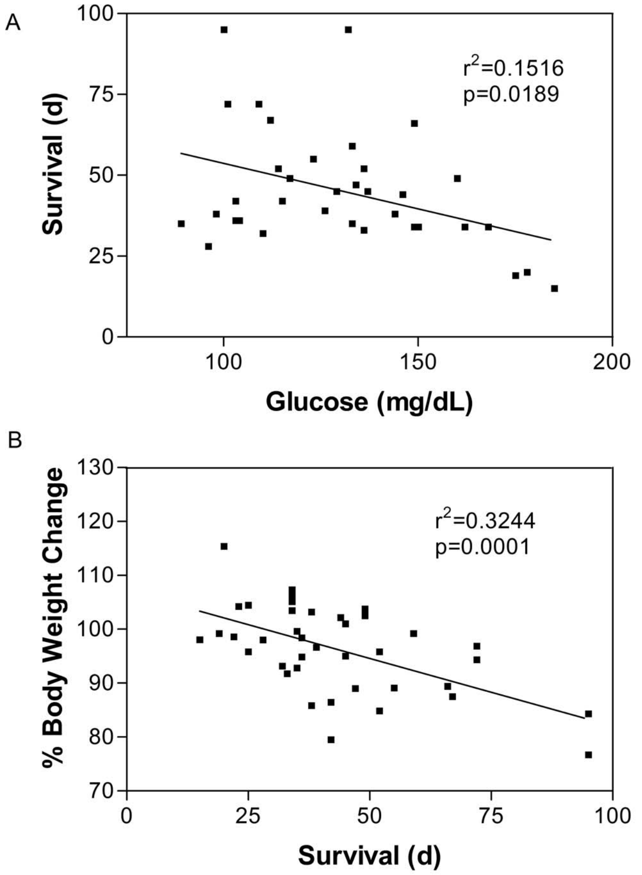

Figure 5. Glucose and weight change are correlated to survival. Linear regression analysis revealed a significant correlation between day 7 blood glucose and percent body weight change with survival ($p = 0.0189$ and $p = 0.0001$, respectively). Results were considered significant when $p < 0.05$.
doi:10.1371/journal.pone.0065522.g005

levels in the KD+HBO_2T mice likely enhanced the efficacy of this combined therapy.

A recent report by Listanti, et. al proposed that tumor-associated fibroblasts produce ketone bodies for cancer cells to use as fuel [68]. The authors have previously published several papers with similar findings [69,70,71]. In these studies, the authors created immortalized fibroblasts which were altered to overexpress rate-limiting enzymes in ketone body production, and co-cultured these cells with human breast cancer cells altered to overexpress enzymes involved in ketone body utilization. While this phenomenon may occur in the genetically altered culture system used by the authors, there is no evidence that this occurs naturally in cancer cells *in vitro* or in tumors *in vivo*. On the contrary, the literature as a whole strongly suggests that cancer cells cannot effectively use ketones for fuel. As described, most cancers do not express the SCOT enzyme which is necessary for ketone body utilization [65,66]. Several studies have reported a deficiency of cancer cells to metabolize ketone bodies *in vitro* [24,25]. Furthermore, it is widely accepted that ketone bodies are produced nearly exclusively from fatty acid β-oxidation in the liver. There is no known metabolic pathway by which fibroblasts can produce ketone bodies from glucose. Without additional compelling evidence to support these claims, we remain proponents of the notion that cancer cells cannot utilize ketone bodies as efficient energy substrates.

Potential concern may arise regarding the use of a diet therapy for cancer patients susceptible to cachexia. While low carbohydrate or ketogenic diets promote weight loss in overweight individuals, they are also known to spare muscle wasting during conditions of energy restriction and starvation [72,73,74,75]. In an animal model of cancer cachexia, administration of a low carbohydrate, high fat diet prevented weight loss of the animals while simultaneously decreasing tumor size [76]. Similar effects were described in human cancer patients [64,77]. The anti-cachexia effects of the KD are not surprising when considering a metabolic switch to fat metabolism and subsequent ketosis evolved as a method of sparing protein during prolonged fasting or starvation [72,78,79]. It makes sense that dietary-induced therapeutic ketosis in a cancer patient would prevent muscle wasting similarly as it does with athletes undergoing intense exercise [80]. Furthermore, when given as an adjuvant treatment to advanced cancer patients, the KD improves quality of life and enhances the efficacy of chemotherapy treatment in the clinic [81,82]. This and other emerging evidence calls into question the common medical advice of limiting fat consumption in overweight cancer patients [83].

Veech and colleagues described the mechanisms by which ketone metabolism protects cells from oxidative damage [74,78], while more recent evidence suggests that ketones function as HDAC inhibitors [84]. βHB metabolism results in an increased reduction of the NAD couple and increased oxidation of the co-enzyme Q inside the mitochondria. Increased oxidation of Q decreases semiquinone levels, subsequently decreasing superoxide anion production [74]. Increased reduction of the NADP couple enhances regeneration of reduced glutathione, an important endogenous antioxidant [74]. Thus, ketone body metabolism protects cells from oxidative damage by decreasing ROS production and by enhancing endogenous antioxidant capabilities. As previously discussed, cancer cells are unable to effectively metabolize ketone bodies; therefore, we do not expect that ketones would confer the same protective effects onto the cancer cell. HBO_2T increases ROS production within the cell which can lead to membrane lipid peroxidation and cell death [85]. Cancer cells with mitochondrial damage and chaotic perfusion naturally produce chronically elevated levels of ROS but are susceptible to oxidative damage-induced cell death with even modest increases in ROS [58,86]. We propose a potential mechanism of KD+HBO_2T efficacy: the KD weakens cancer cells by glucose restriction and the inherent anti-cancer effects of ketone bodies while simultaneously conferring a protective advantage to the healthy tissue capable of ketone metabolism. This metabolic targeting sensitizes the cancer cells to HBO_2T-induced ROS production and oxidative damage, contributing to the efficacy of combining KD with HBO_2T. Additionally, ketone metabolism by the healthy tissues likely confers protection against the potential negative consequences of HBO_2T (CNS oxygen toxicity) [87,88,89]. Recent *in vivo* studies support the neuroprotective effects of ketone esters [90,91]. These hypothetical mechanisms may contribute to the safety and efficacy of the KD+HBO_2T combined therapy.

Stuhr and Moen recently published a comprehensive review of the literature on the use of HBO_2T for cancer [59]. The authors concluded that HBO_2T should be considered a safe treatment for patients with varying malignancies and that there is no convincing evidence its use promotes cancer progression or recurrence. In the literature, there are a substantial number of studies indicating that HBO_2T can induce marked anti-cancer effects *in vitro* and in animal and human studies alike [58,59,92]. Evidence is mixed, however, as other studies reported no effect with HBO_2T [58,59].

Indeed, in our present study, HBO_2T alone did not improve the outcome of VM mice with metastatic cancer, but combining HBO_2T with KD elicited a dramatic therapeutic effect. Perhaps adding the KD or another metabolic therapy (e.g. 2-deoxyglucose, 3-bromopyruvate) to HBO_2T would produce similar results in these previously reported studies demonstrating no efficacy due to HBO_2T alone. It is important to look for synergistic interactions between therapies which may increase the efficacy of cancer treatment. Scheck and coworkers reported complete remission without recurrence in 9 of 11 mice with glioma by combining the KD with radiation [93]. Marsh, et al. reported synergy between the restricted ketogenic diet and the glycolysis inhibitor 2-deoxyglucose [94]. Might adding HBO_2T to these combination therapies elicit even better results? Similarly, might the use of adjuvant therapies like KD and HBO_2T enhance patient response to standard care?

Our study strongly suggests that combining a KD with HBO_2T may be an effective non-toxic therapy for the treatment of metastatic cancer. The efficacy of combining these non-toxic treatments should be further studied to determine their potential for clinical use. Based on the reported evidence, it is highly likely that these therapies would not only contribute to cancer treatment on their own, but might also enhance the efficacy of current standard of care and improve the outcome of patients with metastatic disease.

Acknowledgments

We would like to thank Scivation Inc. for their charitable donation that helped to fund this study.

Author Contributions

Conceived and designed the experiments: AMP TNS DPD. Performed the experiments: AMP CA DPD. Analyzed the data: AMP TNS DPD. Contributed reagents/materials/analysis tools: TNS DPD. Wrote the paper: AMP TNS DPD.

References

1. Gupta G, Massagué J (2006) Cancer metastasis: building a framework. Cell 127: 679–695.
2. Graeme M, Robyn W, Michael B (2004) The contribution of cytotoxic chemotherapy to 5-year survival in adult malignancies. Clinical Oncology 16: 549–560.
3. Sun Y, Campisi J, Higano C, Beer TM, Porter P, et al. (2012) Treatment-induced damage to the tumor microenvironment promotes prostate cancer therapy resistance through WNT16B. Nature medicine 18: 1359–1369.
4. Seyfried TN, Shelton LM, Mukherjee P (2010) Does the existing standard of care increase glioblastoma energy metabolism? The lancet oncology 11: 811–813.
5. Fomchenko E, Holland E (2006) Mouse models of brain tumors and their applications in preclinical trials. Clinical cancer research : an official journal of the American Association for Cancer Research 12: 5288–5297.
6. Seyfried TN (2012) Cancer as a Metabolic Disease: On the Origin, Management, and Prevention of Cancer. Hoboken, NJ: John Wiley & Sons, In. 421 p.
7. Huysentruyt LC, Mukherjee P, Banerjee D, Shelton LM, Seyfried TN (2008) Metastatic cancer cells with macrophage properties: evidence from a new murine tumor model. International journal of cancer 123: 73–84.
8. Huysentruyt LC, Shelton LM, Seyfried TN (2010) Influence of methotrexate and cisplatin on tumor progression and survival in the VM mouse model of systemic metastatic cancer. International journal of cancer Journal international du cancer 126: 65–72.
9. Huysentruyt L, Akgoc Z, Seyfried T (2011) Hypothesis: are neoplastic macrophages/microglia present in glioblastoma multiforme? ASN neuro 3: 183–193.
10. Shelton L, Mukherjee P, Huysentruyt L, Urits I, Rosenberg J, et al. (2010) A novel pre-clinical in vivo mouse model for malignant brain tumor growth and invasion. Journal of neuro-oncology 99: 165–241.
11. Bergamo A, Gagliardi R, Scarcia V, Furlani A, Alessio E, et al. (1999) In vitro cell cycle arrest, in vivo action on solid metastasizing tumors, and host toxicity of the antimetastatic drug NAMI-A and cisplatin. The Journal of pharmacology and experimental therapeutics 289: 559–564.
12. Loehrer P, Einhorn L (1984) Drugs five years later. Cisplatin. Annals of internal medicine 100: 704–713.
13. McGuire WP, Hoskins WJ, Brady MF, Kucera PR, Partridge EE, et al. (1996) Cyclophosphamide and cisplatin versus paclitaxel and cisplatin: a phase III randomized trial in patients with suboptimal stage III/IV ovarian cancer (from the Gynecologic Oncology Group). Seminars in oncology 23: 40–47.
14. Seyfried T, Shelton L (2010) Cancer as a metabolic disease. Nutrition & metabolism 7: 7.
15. Warburg O (1956) On the origin of cancer cells. Science 123: 309–314.
16. Frezza C, Pollard P, Gottlieb E (2011) Inborn and acquired metabolic defects in cancer. Journal of molecular medicine (Berlin, Germany) 89: 213–233.
17. Bayley J-P, Devilee P (2012) The Warburg effect in 2012. Current opinion in oncology 24: 62–69.
18. Duranti L, Leo F, Pastorino U (2012) PET scan contribution in chest tumor management: a systematic review for thoracic surgeons. Tumori 98: 175–184.
19. Katyal NG, Koehler AN, McGhee B, Foley CM, Crumrine PK (2000) The ketogenic diet in refractory epilepsy: the experience of Children's Hospital of Pittsburgh. Clinical pediatrics 39: 153–159.
20. Johnstone A, Horgan G, Murison S, Bremner D, Lobley G (2008) Effects of a high-protein ketogenic diet on hunger, appetite, and weight loss in obese men feeding ad libitum. The American journal of clinical nutrition 87: 44–55.
21. Hussain TA, Mathew TC, Dashti AA, Asfar S, Al-Zaid N, et al. (2012) Effect of low-calorie versus low-carbohydrate ketogenic diet in type 2 diabetes. Nutrition 28: 1016–1021.
22. Volek J, Sharman M, Gómez A, Judelson DA, Rubin M, et al. (2004) Comparison of energy-restricted very low-carbohydrate and low-fat diets on weight loss and body composition in overweight men and women. Nutrition & metabolism 1: 13.
23. Veech R (2004) The therapeutic implications of ketone bodies: the effects of ketone bodies in pathological conditions: ketosis, ketogenic diet, redox states, insulin resistance, and mitochondrial metabolism. Prostaglandins, leukotrienes, and essential fatty acids 70: 309–328.
24. Maurer G, Brucker D, Bähr O, Harter P, Hattingen E, et al. (2011) Differential utilization of ketone bodies by neurons and glioma cell lines: a rationale for ketogenic diet as experimental glioma therapy. BMC cancer 11: 315.
25. Skinner R, Trujillo A, Ma X, Beierle E (2009) Ketone bodies inhibit the viability of human neuroblastoma cells. Journal of pediatric surgery 44: 212.
26. Zhou W, Mukherjee P, Kiebish M, Markis W, Mantis J, et al. (2007) The calorically restricted ketogenic diet, an effective alternative therapy for malignant brain cancer. Nutrition & metabolism 4: 5.
27. Zuccoli G, Marcello N, Pisanello A, Servadei F, Vaccaro S, et al. (2010) Metabolic management of glioblastoma multiforme using standard therapy together with a restricted ketogenic diet: Case Report. Nutrition & metabolism 7: 33.
28. Mavropoulos J, Buschemeyer W, Tewari A, Rokhfeld D, Pollak M, et al. (2009) The effects of varying dietary carbohydrate and fat content on survival in a murine LNCaP prostate cancer xenograft model. Cancer prevention research (Philadelphia, Pa) 2: 557–565.
29. Otto C, Kaemmerer U, Illert B, Muehling B, Pfetzer N, et al. (2008) Growth of human gastric cancer cells in nude mice is delayed by a ketogenic diet supplemented with omega-3 fatty acids and medium-chain triglycerides. BMC cancer 8: 122.
30. Mavropoulos J, Isaacs W, Pizzo S, Freedland S (2006) Is there a role for a low-carbohydrate ketogenic diet in the management of prostate cancer? Urology 68: 15–18.
31. Masko E, Thomas J, Antonelli J, Lloyd J, Phillips T, et al. (2010) Low-carbohydrate diets and prostate cancer: how low is "low enough"? Cancer prevention research (Philadelphia, Pa) 3: 1124–1131.
32. Wheatley KE, Williams EA, Smith NC, Dillard A, Park EY, et al. (2008) Low-carbohydrate diet versus caloric restriction: effects on weight loss, hormones, and colon tumor growth in obese mice. Nutrition and cancer 60: 61–68.
33. Rossifanelli F, Franchi F, Mulieri M, Cangiano C, Cascino A, et al. (1991) Effect of Energy Substrate Manipulation on Tumor-Cell Proliferation in Parenterally Fed Cancer-Patients. Clinical Nutrition 10: 228–232.
34. Tisdale MJ, Brennan RA (1988) A comparison of long-chain triglycerides and medium-chain triglycerides on weight loss and tumour size in a cachexia model. British journal of cancer 58: 580–583.
35. Mukherjee P, El-Abbadi M, Kasperzyk J, Ranes M, Seyfried T (2002) Dietary restriction reduces angiogenesis and growth in an orthotopic mouse brain tumour model. British journal of cancer 86: 1615–1621.
36. Mukherjee P, Sotnikov A, Mangian H, Zhou J, Visek W, et al. (1999) Energy intake and prostate tumor growth, angiogenesis, and vascular endothelial growth factor expression. Journal of the National Cancer Institute 91: 512–523.
37. Thompson H, McGinley J, Spoelstra N, Jiang W, Zhu Z, et al. (2004) Effect of dietary energy restriction on vascular density during mammary carcinogenesis. Cancer research 64: 5643–5650.

38. Hursting S, Smith S, Lashinger L, Harvey A, Perkins S (2010) Calories and carcinogenesis: lessons learned from 30 years of calorie restriction research. Carcinogenesis 31: 83–89.
39. Thompson H, Zhu Z, Jiang W (2003) Dietary energy restriction in breast cancer prevention. Journal of mammary gland biology and neoplasia 8: 133–142.
40. Thompson H, Zhu Z, Jiang W (2004) Identification of the apoptosis activation cascade induced in mammary carcinomas by energy restriction. Cancer research 64: 1541–1545.
41. Zhu Z, Jiang W, McGinley J, Wolfe P, Thompson H (2005) Effects of dietary energy repletion and IGF-1 infusion on the inhibition of mammary carcinogenesis by dietary energy restriction. Molecular carcinogenesis 42: 170–176.
42. Marsh J, Mukherjee P, Seyfried TN (2008) Akt-dependent proapoptotic effects of dietary restriction on late-stage management of a phosphatase and tensin homologue/tuberous sclerosis complex 2-deficient mouse astrocytoma. Clinical cancer research : an official journal of the American Association for Cancer Research 14: 7751–7762.
43. Mukherjee P, Mulrooney T, Marsh J, Blair D, Chiles T, et al. (2008) Differential effects of energy stress on AMPK phosphorylation and apoptosis in experimental brain tumor and normal brain. Molecular cancer 7: 37.
44. Vaupel P, Thews O, Hoeckel M (2001) Treatment resistance of solid tumors: role of hypoxia and anemia. Medical oncology (Northwood, London, England) 18: 243–259.
45. Hoogsteen I, Marres H, van der Kogel A, Kaanders J (2007) The hypoxic tumour microenvironment, patient selection and hypoxia-modifying treatments. Clinical oncology (Royal College of Radiologists (Great Britain)) 19: 385–396.
46. Vaupel P, Mayer A, Höckel M (2004) Tumor hypoxia and malignant progression. Methods in enzymology 381: 335–354.
47. Vaupel P, Harrison L (2004) Tumor hypoxia: causative factors, compensatory mechanisms, and cellular response. The oncologist 9 Suppl 5: 4–9.
48. Gray L, Conger A, Ebert M, Hornsey S, Scott O (1953) The concentration of oxygen dissolved in tissues at the time of irradiation as a factor in radiotherapy. The British journal of radiology 26: 638–648.
49. Wouters B, van den Beucken T, Magagnin M, Lambin P, Koumenis C (2004) Targeting hypoxia tolerance in cancer. Drug resistance updates : reviews and commentaries in antimicrobial and anticancer chemotherapy 7: 25–40.
50. Le Q-T, Denko N, Giaccia A (2004) Hypoxic gene expression and metastasis. Cancer metastasis reviews 23: 293–310.
51. Gill AL, Bell CNA (2004) Hyperbaric oxygen: its uses, mechanisms of action and outcomes. QJM 97.
52. Stuhr L, Raa A, Oyan A, Kalland K, Sakariassen P, et al. (2007) Hyperoxia retards growth and induces apoptosis, changes in vascular density and gene expression in transplanted gliomas in nude rats. Journal of neuro-oncology 85: 191–393.
53. Moen I, Øyan A, Kalland K-H, Tronstad K, Akslen L, et al. (2009) Hyperoxic treatment induces mesenchymal-to-epithelial transition in a rat adenocarcinoma model. PloS one 4.
54. Stuhr L, Iversen V, Straume O, Maehle B, Reed R (2004) Hyperbaric oxygen alone or combined with 5-FU attenuates growth of DMBA-induced rat mammary tumors. Cancer letters 210: 35–75.
55. Bennett M, Feldmeier J, Smee R, Milross C (2008) Hyperbaric oxygenation for tumour sensitisation to radiotherapy: a systematic review of randomised controlled trials. Cancer treatment reviews 34: 577–591.
56. Takiguchi N, Saito N, Nunomura M, Kouda K, Oda K, et al. (2001) Use of 5-FU plus hyperbaric oxygen for treating malignant tumors: evaluation of antitumor effect and measurement of 5-FU in individual organs. Cancer chemotherapy and pharmacology 47: 11–14.
57. Petre P, Baciewicz F, Tigan S, Spears J (2003) Hyperbaric oxygen as a chemotherapy adjuvant in the treatment of metastatic lung tumors in a rat model. The Journal of thoracic and cardiovascular surgery 125: 85.
58. Daruwalla J, Christophi C (2006) Hyperbaric oxygen therapy for malignancy: a review. World journal of surgery 30: 2112–2143.
59. Moen I, Stuhr LE (2012) Hyperbaric oxygen therapy and cancer-a review. Targeted oncology 7: 233–242.
60. Scheck A, Abdelwahab M, Fenton K, Stafford P (2011) The ketogenic diet for the treatment of glioma: Insights from genetic profiling. Epilepsy research 100: 327–337.
61. Astrup A, Ryan L, Grunwald G, Storgaard M, Saris W, et al. (2000) The role of dietary fat in body fatness: evidence from a preliminary meta-analysis of ad libitum low-fat dietary intervention studies. The British journal of nutrition 83: S25–32.
62. Fine E, Segal-Isaacson C, Feinman R, Herszkopf S, Romano M, et al. (2012) Targeting insulin inhibition as a metabolic therapy in advanced cancer: a pilot safety and feasibility dietary trial in 10 patients. Nutrition 28: 1028–1035.
63. Seyfried T, Sanderson T, El-Abbadi M, McGowan R, Mukherjee P (2003) Role of glucose and ketone bodies in the metabolic control of experimental brain cancer. British journal of cancer 89: 1375–1457.
64. Nebeling LC, Miraldi F, Shurin SB, Lerner E (1995) Effects of a ketogenic diet on tumor metabolism and nutritional status in pediatric oncology patients: two case reports. Journal of the American College of Nutrition 14: 202–208.
65. Tisdale M, Brennan R (1983) Loss of acetoacetate coenzyme A transferase activity in tumours of peripheral tissues. British journal of cancer 47: 293–297.
66. Sawai M, Yashiro M, Nishiguchi Y, Ohira M, Hirakawa K (2004) Growth-inhibitory effects of the ketone body, monoacetoacetin, on human gastric cancer cells with succinyl-CoA: 3-oxoacid CoA-transferase (SCOT) deficiency. Anticancer research 24: 2213–2217.
67. Magee BA, Potezny N, Rofe AM, Conyers RA (1979) The inhibition of malignant cell growth by ketone bodies. The Australian journal of experimental biology and medical science 57: 529–539.
68. Martinez-Outschoorn UE, Lin Z, Whitaker-Menezes D, Howell A, Sotgia F, et al. (2012) Ketone body utilization drives tumor growth and metastasis. Cell cycle 11: 3964–3971.
69. Martinez-Outschoorn U, Prisco M, Ertel A, Tsirigos A, Lin Z, et al. (2011) Ketones and lactate increase cancer cell "stemness," driving recurrence, metastasis and poor clinical outcome in breast cancer: achieving personalized medicine via Metabolo-Genomics. Cell cycle 10: 1271–1286.
70. Bonuccelli G, Tsirigos A, Whitaker-Menezes D, Pavlides S, Pestell R, et al. (2010) Ketones and lactate "fuel" tumor growth and metastasis: Evidence that epithelial cancer cells use oxidative mitochondrial metabolism. Cell cycle 9: 3506–3514.
71. Salem A, Whitaker-Menezes D, Lin Z, Tanowitz H, Al-Zoubi M, et al. (2012) Two-compartment tumor metabolism: Autophagy in the tumor microenvironment and oxidative mitochondrial metabolism (OXPHOS) in cancer cells. Cell cycle 11: 2545–2601.
72. Manninen AH (2006) Very-low-carbohydrate diets and preservation of muscle mass. Nutrition & metabolism 3: 9.
73. Cahill G (2006) Fuel metabolism in starvation. Annual review of nutrition 26: 1–22.
74. Veech RL (2004) The therapeutic implications of ketone bodies: the effects of ketone bodies in pathological conditions: ketosis, ketogenic diet, redox states, insulin resistance, and mitochondrial metabolism. Prostaglandins, leukotrienes, and essential fatty acids 70: 309–319.
75. Volek J, Sharman M, Love D, Avery N, Gómez A, et al. (2002) Body composition and hormonal responses to a carbohydrate-restricted diet. Metabolism: clinical and experimental 51: 864–870.
76. Tisdale MJ, Brennan RA, Fearon KC (1987) Reduction of weight loss and tumour size in a cachexia model by a high fat diet. British journal of cancer 56: 39–43.
77. Nebeling LC, Lerner E (1995) Implementing a ketogenic diet based on medium-chain triglyceride oil in pediatric patients with cancer. Journal of the American Dietetic Association 95: 693–697.
78. Veech RL, Chance B, Kashiwaya Y, Lardy HA, Cahill GF, Jr. (2001) Ketone bodies, potential therapeutic uses. IUBMB life 51: 241–247.
79. Wu GY, Thompson JR (1988) The effect of ketone bodies on alanine and glutamine metabolism in isolated skeletal muscle from the fasted chick. The Biochemical journal 255: 139–144.
80. Paoli A, Grimaldi K, D'Agostino D, Cenci L, Moro T, et al. (2012) Ketogenic diet does not affect strength performance in elite artistic gymnasts. Journal of the International Society of Sports Nutrition 9: 34.
81. Stafford P, Abdelwahab M, Kim DY, Preul M, Rho J, et al. (2010) The ketogenic diet reverses gene expression patterns and reduces reactive oxygen species levels when used as an adjuvant therapy for glioma. Nutrition & metabolism 7: 74.
82. Schmidt M, Pfetzer N, Schwab M, Strauss I, Kämmerer U (2011) Effects of a ketogenic diet on the quality of life in 16 patients with advanced cancer: A pilot trial. Nutrition & metabolism 8: 54.
83. Champ C, Volek J, Siglin J, Jin L, Simone N (2012) Weight gain, metabolic syndrome, and breast cancer recurrence: are dietary recommendations supported by the data? International journal of breast cancer 2012: 506868.
84. Shimazu T, Hirschey MD, Newman J, He W, Shirakawa K, et al. (2013) Suppression of oxidative stress by beta-hydroxybutyrate, an endogenous histone deacetylase inhibitor. Science 339: 211–214.
85. D'Agostino D, Olson J, Dean J (2009) Acute hyperoxia increases lipid peroxidation and induces plasma membrane blebbing in human U87 glioblastoma cells. Neuroscience 159: 1011–1033.
86. Aykin-Burns N, Ahmad I, Zhu Y, Oberley L, Spitz D (2009) Increased levels of superoxide and H2O2 mediate the differential susceptibility of cancer cells versus normal cells to glucose deprivation. The Biochemical journal 418: 29–66.
87. D'Agostino D, Pilla R, Held H, Landon CS, Ari C, et al. (2012) Development, testing, and therapeutic applications of ketone esters (KE) for CNS oxygen toxicity (CNS-OT); i.e., hyperbaric oxygen (HBO2)-induced seizures. FASEB. San Diego, CA.
88. Pilla R, D'Agostino D, Landon C, Dean J (2012) Intragastric ketone esters administration prevents central nervous system oxygen toxicity via tidal volume and respiratory frequency modulation in rats. Third International Symposium on Dietary Therapies for Epilepsy & Other Neurological Disorders.
89. Bennett A, Ari C, Kesl S, Luke J, Diamond D, et al. (2012) Effect of ketone treatment and glycolysis inhibition in brain cancer cells (U87MG) and rat primary cultured neurons exposed to hyperbaric oxygen and amyloid beta. FASEB J.
90. D'Agostino DP, Pilla R, Held HE, Landon CS, Puchowicz M, et al. (2013) Therapeutic ketosis with ketone ester delays central nervous system oxygen toxicity seizures in rats. American journal of physiology Regulatory, integrative and comparative physiology.
91. Kashiwaya Y, Bergman C, Lee JH, Wan R, King MT, et al. (2013) A ketone ester diet exhibits anxiolytic and cognition-sparing properties, and lessens amyloid and tau pathologies in a mouse model of Alzheimer's disease. Neurobiology of aging 34: 1530–1539.

92. Al-Waili NS, Butler GJ, Beale J, Hamilton RW, Lee BY, et al. (2005) Hyperbaric oxygen and malignancies: a potential role in radiotherapy, chemotherapy, tumor surgery and phototherapy. Medical science monitor : international medical journal of experimental and clinical research 11: RA279–289.
93. Abdelwahab M, Fenton K, Preul M, Rho J, Lynch A, et al. (2012) The ketogenic diet is an effective adjuvant to radiation therapy for the treatment of malignant glioma. PloS one 7.
94. Marsh J, Mukherjee P, Seyfried T (2008) Drug/diet synergy for managing malignant astrocytoma in mice: 2-deoxy-D-glucose and the restricted ketogenic diet. Nutrition & metabolism 5: 33.
